# Halophilic Microorganisms Are Responsible for the Rosy Discolouration of Saline Environments in Three Historical Buildings with Mural Paintings

**DOI:** 10.1371/journal.pone.0103844

**Published:** 2014-08-01

**Authors:** Jörg D. Ettenauer, Valme Jurado, Guadalupe Piñar, Ana Z. Miller, Markus Santner, Cesareo Saiz-Jimenez, Katja Sterflinger

**Affiliations:** 1 VIBT-BOKU, University of Natural Resources and Life Sciences, Department of Biotechnology, Vienna, Austria; 2 Instituto de Recursos Naturales y Agrobiologia, IRNAS-CSIC, Sevilla, Spain; 3 CEPGIST/CERENA, Instituto Superior Técnico, Universidade de Lisboa, Lisboa, Portugal; 4 Bundesdenkmalamt, Abteilung für Konservierung und Restaurierung, Vienna, Austria; University Hospital of the Albert-Ludwigs-University Freiburg, Germany

## Abstract

A number of mural paintings and building materials from monuments located in central and south Europe are characterized by the presence of an intriguing rosy discolouration phenomenon. Although some similarities were observed among the bacterial and archaeal microbiota detected in these monuments, their origin and nature is still unknown. In order to get a complete overview of this biodeterioration process, we investigated the microbial communities in saline environments causing the rosy discolouration of mural paintings in three Austrian historical buildings using a combination of culture-dependent and -independent techniques as well as microscopic techniques. The bacterial communities were dominated by halophilic members of Actinobacteria, mainly of the genus Rubrobacter. Representatives of the Archaea were also detected with the predominating genera Halobacterium, Halococcus and Halalkalicoccus. Furthermore, halophilic bacterial strains, mainly of the phylum Firmicutes, could be retrieved from two monuments using special culture media. Inoculation of building materials (limestone and gypsum plaster) with selected isolates reproduced the unaesthetic rosy effect and biodeterioration in the laboratory.

## Introduction

It is well-known that microorganisms play a crucial role in the degradation and deterioration of mural paintings and building materials. Stone materials and wall paintings provide a great variety of ecological niches for all types of microorganisms that can induce biodeterioration. Biodegradation is caused by biochemical processes, through bio-corrosion, dissolution and solubilization of material components, however aesthetical effects are often more evident in some biodeterioration processes. The aesthetical changes are triggered by the deterioration of painting pigments on walls and/or by the formation of coloured biofilms or excretion of extracellular pigments. Fungi, algae, different bacteria and archaea produce a wide variety of biogenic pigments such as chlorophyll, carotenes, phenols, anthraquinones and melanin with colours ranging from light yellow, orange, pink, purple, violet, green, grey, dark brown to black [1]–[5]. The formation of orange to red pigments is due to the production of carotenes as a means of protecting the cells against high UV-radiation, chemical- and/or salt stress [6]. On salty walls the inhabiting halophilic bacteria and haloarchaea usually form pink to purple or violet stains. Orange pigmentations on sandstone or marble often resemble iron oxide and therefore it has to be clarified if the discolouration is due to microbial growth [7], [8]. The biogenic pigments are usually very stable on the materials even if the causative microorganisms are already dead.

**Figure 1:**
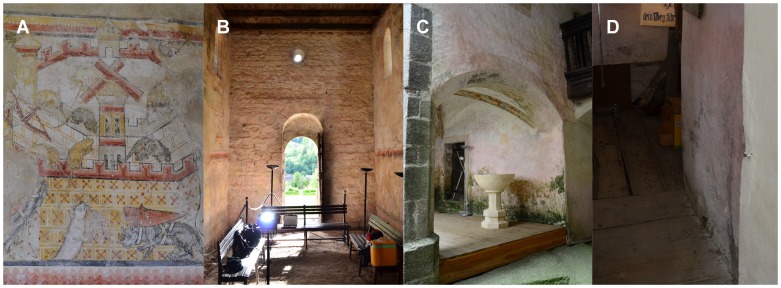
The three historical buildings. The Johannes Chapel in Pürgg (A, B), the castle Rappottenstein (C) and the Saint Rupert Chapel in Weißpriach (D): A) The famous romanesque fresco „Cats-mice-war“. B) Interior view onto the West-wall in the Johannes chapel in Pürgg which was totally covered with a rosy biofilm. C) Differently coloured biofilms cover the inner courtyard of the castle Rappottenstein with rosy strains spreading upwards. D) Pink discolouration of the wall in the tower-room of the Saint Rupert chapel in Weißpriach. All photographs were taken by Jörg D. Ettenauer.

In this study we investigated the pink to rosy discolouration phenomenon presented by two historical chapels and a medieval castle located in Austria (Figure S1). The Johannes Chapel in Pürgg (Styria) was built in Romanesque style and dates back to the 12th century. The frescos inside with the famous and mysterious motive of the “cats-mice-war” was one of the most prominent and well-preserved Romanesque paintings in Europe, dated to 1160 (Figure 1A). After constructional changes on the whole west wall a rosy biofilm established which further spread across the whole chapel (Figure 1B).

The castle of Rappottenstein (Lower Austria) is a medieval castle, which was never conquered and is, therefore, one of the most well-preserved castles in Austria. It dates back to 12th century and combines three constructional ages with correspondent wall paintings: Romanesque, Gothic and Renaissance. The walls on the ground floor and the inner courtyard are covered with green to black biofilms but also a strong rosy discolouration of the walls can be observed that spreads to the inside of the rooms on the ground- and further up till the second floor (Figure 1C).

The Saint Rupert chapel of Weißpriach, in Salzburg is the oldest of the three buildings and dates back to 750 AD. The walls contain Romanesque decorative stones and many outstanding frescos that date back from the 10th to 13th century. Due to a leaky roof, water caused the formation of salt efflorescences and a strong rosy pigmentation can be found in the tower room (Figure 1D). During the last years the pigmentation extended into the actual chapel.

All three buildings are exposed to harsh climatic conditions due to their geographical location in alpine regions and/or their local site. Hence, all of them suffer from different types of water impact: i) water mostly enters the buildings from outside through the ground into the walls and ii) additionally water from rain and snow directly enters the buildings through a leaky roof, open windows or directly into the inner courtyard, respectively. As a result, a variety of hygroscopic, soluble salts in the porous buildings materials and wall paintings are solubilized and further migrate with the capillary water through the stone. The changes in the physical parameters lead to crystallization of the salts at the surfaces, the so-called salt efflorescences. Furthermore, the water impact and crystallization of salts cause a physical stress for the materials leading to cracking, powdering and flaking of the surface material and material loss [9].

Several reports have shown that ancient stone works and mural paintings represent a common habitat for extremely salt tolerant and moderate halophilic bacteria [10]–[13] and archaea [10], [14]. The rosy discolouration of stone works in different historical buildings located in various parts of Europe was also already subject of different studies [3], [15]–[17].

The objective of this work was the identification of the microorganisms present in the red to pink pigmented sites of the three buildings, in order to compare the data with those obtained in previous investigations on other monuments situated in different geographical locations of Europe. Finally, the results should be a basis to advice conservators in order to protect the buildings from further biodeterioration.

To analyse the microbial communities inhabiting the wall surface of the three buildings, samples were collected from different sites of each building where a rosy discolouration could be observed. Thereafter, to evaluate the whole structure of the microbial communities inhabiting the walls, three strategies were applied: Classical cultivation techniques, to identify the cultivable members of the microbial communities, molecular techniques - comprising direct DNA extraction, PCR amplification of ribosomal 16S rRNA gene sequences, DGGE analysis and construction and screening of clone libraries - to identify the non-cultivable fraction of the communities, and reproduction of the phenomenon in the laboratory using selected isolates and building materials.

## Materials and Methods

### Sampling

Stone samples with rosy biofilms (about 0.16 to 14.52 g) were taken from 4–16 different locations of each building, hereafter referred to as ‘P’-samples from Pürgg, ‘W’-samples from Weißpriach and ‘R’-samples from Rappottenstein, respectively (Table S1). The samples were collected with sterile scalpels by scraping off wall material to a depth of 1–3 mm or by taking partly detached plaster pieces of 1–4 cm^2^ from the walls using sterile forceps, respectively. Each aseptically collected sample was immediately stored in sterile plastic tubes or containers, respectively, and transported under cooled conditions to the laboratory for further analysis. The samplings were performed under the supervision of different conservators due to the high value of the objects. Permissions for sampling at each location were issued by the Ministry of Care of Monuments in Austria (Bundesdenkmalamt) as well as the local municipal office and church, respectively. Stone samples from similar locations of each building were mixed and divided into two parts. In the case of Pürgg the pooled samples were called P1, P2 and P3. Regarding the Saint Rupert chapel of Weißpriach, the mingled samples were named W1, W2 and W3 and R1, R2 and R3 for the castle of Rappottenstein, respectively (Table S1). The larger proportion of each sample (1.4–10 g) was sent under cooled conditions to Spain for cultivation analyses and the smaller part was used for molecular analyses.

### Cultivation analysis

All samples were inoculated at 28°C for 30 days on different culture media: trypticase soy agar supplemented with Na and Mg (TSA Na-Mg [18], TSA Na-Mg supplemented with 15% NaCl (w/v) instead of 3% NaCl (w/v), nutrient agar (Difco, Becton Dickinson, Sparks MD, USA) diluted 1∶100, nutrient agar supplemented with NaCl (3%, w/v), marine agar 2216 (Difco, Becton Dickinson, Sparks MD, USA), DSMZ media 372 (http://www.dsmz.de/microorganisms/medium/pdf/DSMZ_Medium372.pdf) and 1018 (http://www.dsmz.de/microorganisms/medium/pdf/DSMZ_Medium1018.pdf). The last two culture media were specific for Archaea. All orange or pink pigmented colonies that might be responsible for the aesthetical damage of the objects were picked up and transferred to fresh medium.

### Molecular characterization of the isolated strains

Bacterial DNA was extracted following the method described by Marmur [19]. The 16S rRNA gene was amplified by PCR using the conserved primers 27F [20] and 1522R [21] with the following PCR thermal conditions: 95°C for 1 min; 35 cycles of 95°C for 15 s, 55°C for 15 s, 72°C for 2 min; and a final extension cycle at 72°C for 10 min. Forward and reverse strands of the amplified DNA fragment were sequenced in an ABI 3700 sequencer (Applied Biosystems). The identification of phylogenetic neighbours was carried out by the BLAST program [22] against the NCBI database and the database of type strains EZtaxon [23] with validly published prokaryotic names.

### Molecular analysis - DNA extraction from the walls

The mingled samples of each building were ground for 2 minutes in liquid nitrogen using a sterile mortar and pestle. From the homogenized material each 100 mg were weighed in a Sartorius precision scale for DNA extraction. The complete microbial DNA was directly isolated using the FastDNA SPIN Kit for soil from MP Biomedicals (Illkrich, France). The DNA concentration, -quality and -purity, respectively, was measured using a NanoDrop ND-1000 spectrophotometer (peqLabBiotechnologie GmbH, Linz, Austria). Additionally, the extracted DNA was visualized on 1.5 (w/v) agarose gels at 110V for 40 minutes, stained in an ethidium bromide solution [1 µg ml^−1^; stock: 10 mg ml^−1^] for 20 minutes and documented using an UVP documentation system (BioRad Transilluminator, Universal Hood; Mitsubishi P93D-printer).

### PCR amplification of bacterial 16S rDNA fragments

All PCR reactions were executed in a BioRad C1000 Thermal Cycler. The 2× PCR Master Mix (Promega, Mannheim, Germany) [50 units ml^−1^ of TaqDNA Polymerase in a supplied reaction buffer (pH 8.5), 400 µM dATP, 400 µM dGTP, 400 µM dCTP, 400 µM dTTP, 3 mM MgCl_2_] was diluted to 1×, 12.5 pmol of each primer and 2.5 µl of template DNA were added to 25 µl total reaction volumes.

For genetic fingerprinting of the eubacterial 16S rDNA fragments by DGGE two different PCR reactions were performed. For the first round the universal primers 341f [24] and 907r [25] were used. The second round, a semi-nested PCR for genetic fingerprints, was done using the primers 341GC and 518r [26]. The forward primer possesses a 40-base Guanine-Cytosine (GC) clamp at its 5′ end that stabilizes the melting behaviour of the DNA fragments in DGGE analysis [24]. The semi-nested PCR was executed in 100 µl volumes, separated into two tubes to which each 50 µl mastermix, 25 pmol of each primer and 3.5 µl of template were added. The thermocycling conditions described by Schabereiter-Gurtner et al. [27] were used for genetic fingerprinting. Seven microliter of each PCR product was electrophoresed on a 2% (w/v) agarose gel as described above. In each PCR reaction a negative control was included, where no DNA template was added, to exclude the possibility of cross-contamination.

### PCR amplification of archaeal 16S rDNA fragments

The amplification of archaeal 16S rDNA fragments was carried out similar to the bacterial PCR analysis with the addition of BSA (25 pmol in 25 µl reaction volume) to the mastermix. For the first round the primers ARC344 and ARC915 [28] were applied, using the thermocycling program described by Piñar et al. [29]. To obtain genetic fingerprints a semi-nested PCR was performed with primers 518r carrying a GC clamp at its 5′ end [24] and the archaea specific primer ARC344. The same cycling conditions were used as described for the amplification of the bacterial 16S rDNA.

### Fingerprint analysis by DGGE – Denaturing Gradient Gel Electrophoresis

For DGGE fingerprinting 100 µl PCR products from the semi-nested PCR were pooled, precipitated overnight with 96% ethanol at −20°C and re-suspended in 20 µl ultra-pure water (Qiagen GmbH, Hilden, Germany). The concentrated PCR products supplemented with 5 µl 6× Loading Dye Solution (Thermo Scientific) were separated on gels in 0.5× TAE buffer [20 mM Tris, 10 mM acetate, 0.5 mM Na_2_EDTA; pH 8.0] for 3.5 hours at 200 V and 60°C in a Bio-Rad-DCode – Universal Mutation Detection System [24]. A linear chemical gradient ranging from 35 to 55% of urea and formamide in an 8% (w/v) polyacrylamide gel (Bio-Rad, Munich, Germany) for screening of bacterial communities and from 35 to 50% for separation of bands of the archaeal population was used. After completion of electrophoresis staining of the gels was done in an ethidium bromide solution for 20 minutes and afterwards visualized by a UVP documentation system.

### Construction of 16S rDNA clone libraries and screening by DGGE

In order to obtain phylogenetic identification data on the inhabiting microorganisms, two clone libraries of each sample containing the bacterial or the archaeal 16S rDNA fragments, respectively, were created. Therefore, 2×3.5 µl DNA templates of each sample were amplified in 2×50 µl reaction volumes using the primers for the first round as mentioned above. Aliquots of the PCR products were electrophoresed, purified using the QIAquick PCR Purification Kit (Qiagen GmbH, Hilden, Germany) and re-suspended in 30 µl ultra-pure water. The purified DNA was again analysed by gel electrophoresis and 5.5 µl were used as a ligation template for the pGEM-T easy Vector system (Promega). The ligation products were transformed into One Shot TOP10 cells (Invitrogen, Carlsbad, USA) according to the manufacturer’s instructions. Recombinant cells (white colonies) could be identified on indicator LB medium containing ampicilline (100 µg ml^−1^), streptomycine (25 µg ml^−1^) and X-Gal (5-bromo-4-chloro-3-indolyl-β-D-galactopyranoside; 0.1 mM) [30].

About 50–150 white colonies from each clone library were harvested and screened by DGGE as described by Schabereiter-Gurtner et al. [27]. The band positions of the clones were compared with the DGGE fingerprint of the original sample and inserts of clones matching dominant- and faint bands of the banding profile of the original sample were selected for sequencing.

### 16S rDNA sequencing and sequence analysis

In 2×50 µl reaction volumes with each 3 µl template DNA of the clone inserts were amplified using the vector specific primers SP6 and T7 (Promega, Mannheim, Germany) [27]. After visualization on agarose gels and purification of the pooled PCR products, 25 µl aliquots were sent to GATC Biotech sequencing service (www.gatc-biotech.com). Comparative sequence analysis was done by comparing pair-wise insert sequences with those available in the online databases provided by the NCBI (National Centre for Biotechnology Information), and RDP (Ribosomal Database Project), respectively, using the search program BLAST [22]. The ribosomal sequences of the bacterial- and archaeal clones and the bacterial isolates have been deposited at the NCBI GenBank database under the accession numbers (KF692550–KF692709 for the cloned sequences and HG515390–HG515401 for the bacterial isolates) listed in Tables S2, S3 and S4, for each 16S rDNA sequence.

### Laboratory-based colonization experiment of building materials

Strains of *Halobacillus naozhouensis* and *Kocuria polaris* were used for inoculation and reproduction of biodeterioration processes in the laboratory. Gypsum plasterand Hontoria limestone [31] were sliced into squares of 3×3×0.5 cm, sterilized in an autoclave under fluent vapor and inoculated with suspensions of each bacterium. Probes of gypsum plaster and limestone were inoculated with three different suspensions of cells (A: *Halobacillus naozhouensis*, B: *Kocuria polaris* and C: mixture of both strains) at concentrations of 1.5×10^9^ cells ml−1. All probes were inoculated with 150 µL of suspension and incubated at 30°C for one month.

### Field Emission Scanning Electron Microscopy (FESEM)

FESEM was used to accurately assess surface topography, microbial growth and biodeterioration phenomena on the inoculated gypsum plaster and limestone probes. Bulk fragments were directly mounted on a sample stub and sputter coated with a thin gold/palladium film. Subsequently, samples were examined on a Jeol JSM-7001F microscope equipped with an Oxford X-ray energy dispersive spectroscopy (EDS) detector. FESEM examinations were operated in secondary electron (SE) detection mode with an acceleration potential of 15 kV.

## Results

### Phylogenetic identification of the cultivated microorganisms

During the first 48 hours of incubation up to a maximum of 12 days, twenty-nine bacterial strains with yellow to orange or pink appearance were isolated. Coloured bacterial strains isolated from sample P2 of the Johannes Chapel in Pürgg represented 41.4% of all isolated strains, and, on average, from all three samples of the castle Rappottenstein 19.5%. No cultivable bacteria were found on samples from Weißpriach, which might be due to the known difficulties in culturing these pigmented, halophilic microorganisms and perhaps also due to the low number of samples as well as very small sample amounts that could be taken from this location (Table S1). Similarly, no archaea could be isolated from none of the samples. The molecular identification of the isolated strains is shown in Table 1 and also in Table S2 where the detailed information about each isolate, its closest related neighbour, the isolation source as well as the accession number for the submitted 16S rDNA sequence are given. According to the NCBI database, the bacterial strains showed similarity values ranging from 98 to 100% and could be grouped to cultured members of three different bacterial phyla, namely the Firmicutes (89.7% of all isolated strains), the Proteobacteria (6.9%) and the Actinobacteria (3.5%).

**Table 1:** Phylogenetic analysis.

Culture-independent analysis						
Bacteria		Samples - Building	Pürgg	Rappottenstein	Weißpriach	Total
Phylum	Phylogenetic group	Genus				
**Firmicutes**	Bacillales					**3 (2.8%)**
		*Natribacillus*		1 (2.9%)		*1 (0.9%)*
		*Planococcus*		1 (2.9%)		*1 (0.9%)*
		*Bacillus*		1 (2.9%)		*1 (0.9%)*
**Proteobacteria**						**2 (1.9%)**
	β-Proteobacteria	*Ralstonia*		1 (2.9%)		*1 (0.9%)*
	γ-Proteobacteria	Uncultured Proteobacteria		1 (2.9%)		*1 (0.9%)*
**Actinobacteria**						**72 (67.3%)**
	Rubrobacteridae					*44 (41.1%)*
		Rubrobacteracea*/Rubrobacter*	8 (19.5%)	7 (20%)	8 (25.8%)	*23 (21.5%)*
		Uncultured Rubrobacteridae	13 (31.7%)	5 (14.3%)	3 (9.7%)	*21 (19.6%)*
		Uncultured Actinobacteria	8 (19.5%)	4 (11.4%)	6 (19.4%)	*18 (16.8%)*
	Actinobacteridae	*Actinomycetospora*	1 (2.4%)			*1 (0.9%)*
		*Saccharopolyspora*		1 (2.9%)	1 (3.2%)	*2 (1.9%)*
		*Thermocrispum*		1 (2.9%)		*1 (0.9%)*
		*Amycolatopsis*	1 (2.4%)			*1 (0.9%)*
		*Pseudonocardia*		1 (2.9%)		*1 (0.9%)*
		*Nocardioides*	1 (2.4%)			*1 (0.9%)*
		*Jiangella*		1 (2.9%)		*1 (0.9%)*
		*Nesterenkonia*	1 (2.4%)			*1 (0.9%)*
		*Janibacter*		1 (2.9%)		*1 (0.9%)*
Unclassified bacterium clone			8 (19.5%)	9 (25.7%)	13 (41.9%)	30 (28%)
**Total no. of clones**			**41 (100%)**	**35 (100%)**	**31 (100%)**	**107 (100%)**
**Archaea**		**Samples - Building**	**Pürgg**	**Rappottenstein**	**Weißpriach**	**Total**
Phylum	Family	Genus				
**Euryarchaeota**						**39 (73.6%)**
	Halobacteriaceae					38 (71.7%)
		*Halococcus*	9 (42.9%)	6 (33.3%)	6 (42.9%)	*21 (39.6%)*
		*Halobacterium*	5 (23.8%)	2 (11.1%)	2 (14.3%)	*9 (17%)*
		*Halalkalicoccus*		5 (27.8%)	2 (14.3%)	*7 (13.2%)*
		*Natronorubrum*		1 (5.6%)		*1 (1.9%)*
	Order Halobacteriales	*Haloarchaeon Nie 13*	1 (4.8%)			1 (1.9%)
Unclassified archaeon clone			6 (28.6%)	4 (22.2%)	4 (28.6%)	14 (26.4%)
**Total no. of clones**			**21 (100%)**	**18 (100%)**	**14 (100%)**	**53 (100%)**
**Culture-dependent analysis**						
**Bacteria**		**Samples - Building**	**Pürgg**	**Rappottenstein**	**Weißpriach**	**Total**
Phylum	Family	Genus				
**Firmicutes**	Bacillales					**26 (89.7%)**
		*Halobacillus*	10 (83.3%)	5 (29.4%)		*15 (51.7%)*
		*Planococcus*	2 (16.7%)	4 (23.5%)		*6 (20.7%)*
		*Marinococcus*		3 (17.6%)		*3 (10.3%)*
		*Planomicrobium*		2 (11.8%)		*2 (6.9%)*
**Proteobacteria**						**2 (6.9%)**
	α-Proteobacteria	*Paracoccus*		1 (5.9%)		*1 (3.5%)*
	γ-Proteobacteria	*Halomonas*		1 (5.9%)		*1 (3.5%)*
**Actinobacteria**						**1 (3.5%)**
	Actinomycetales	*Kocuria*		1 (5.9%)		*1 (3.5%)*
**Total no. of isolates**			**12 (100%)**	**17 (100%)**		**29 (100%)**

The results from the culture-independent and -dependent analysis of the samples of the three buildings are shown according the NCBI database search. Number of clones/isolates and calculated percentages (rounded values) indicate the amounts of the 16S rDNA sequences related to the corresponding phylum, phylogenetic group and genera, respectively.

Out of the twelve isolates obtained from sample P2, two isolates from Pürgg were closely affiliated with *Planococcus salinarum* (98%) and the other bacterial strains were highly related to *Halobacillus herbersteinensis* (99%) - all belonging to the phylum Firmicutes (Table S2).

From the castle of Rappottenstein seventeen isolates could be grown on the used culture media: ten bacteria from sample R1, six from sample R2 and one from sample R3. Five isolates from sample R1 showed the best match in the NCBI database search with *Halobacillus herbersteinensis* (99%). Further, three bacteria were affiliated with *Marinococcus luteus* (99%) and one bacterium with *Paracoccus marcusii* (100%), - being all representatives of the phylum Firmicutes. Only one strain showed to be related to members of the Proteobacteria, namely to *Halomonas muralis* (98%). Out of the six cultured bacteria from sample R2, five isolates affiliated also with members of the Firmicutes. Two of them were related to cultivable *Planomicrobium flavidum* (98%), another one to *Planococcus psychrotoleratus* (99%), one to *Planococcus antarcticus* (99%) and one to *Planococcus donghaensis* (99%). The last strain of sample R2 was the only member of the Actinobacteria, which could be identified as *Kocuria rosea* (99%). The only isolate obtained from sample R3 was similar to *Planococcus psychrotoleratus* (99%) belonging to the Firmicutes phylum (Table S2).

Alternatively, using EZtaxon (a 16S rRNA gene sequence database of type strains), NCBI affiliations changed and *Halobacillus herbersteinensis* was identified as *Halobacillus naozhouensis*, *Marinococcus luteus* as *Marinococcus tarijensis*, *Planococcus psychrotoleratus* either as *Planococcus okeanokoites* or *Planococcus donghaensis*, *Planococcus antarcticus* as *Planococcus donghaensis*, and *Kocuria rosea* as *Kocuria polaris* (Table S2).

### Phylogenetic identification of the microbial communities using molecular techniques

**Figure 2:**
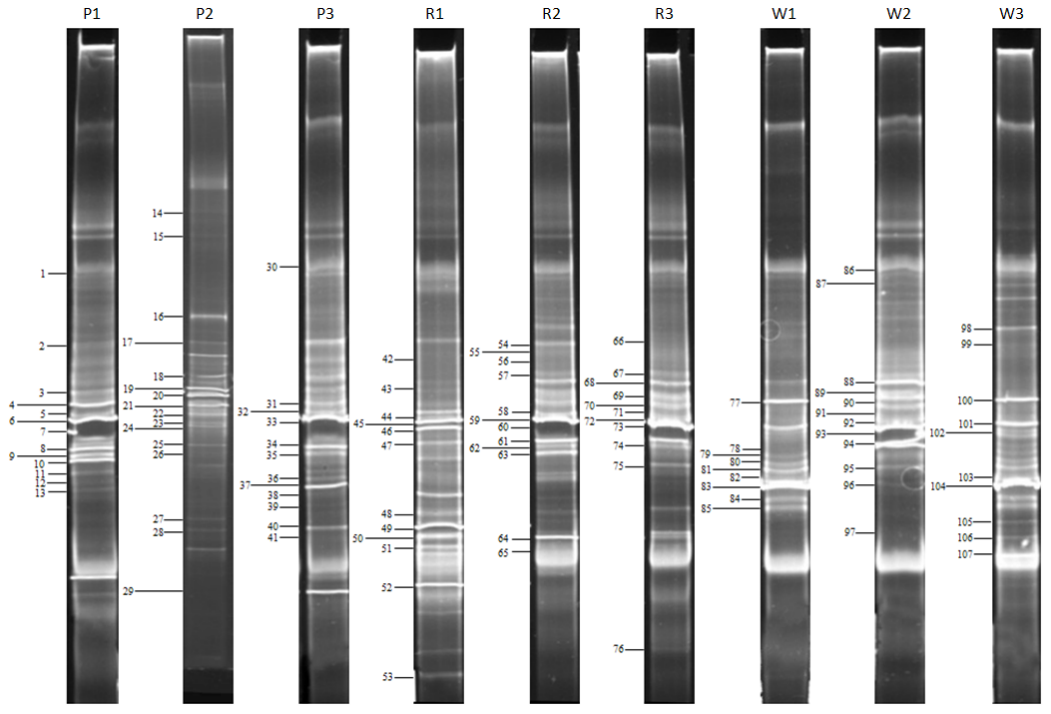
DGGE analysis of the bacterial communities present in the samples from the three buildings. The selected clone sequences are marked with bars, numbered consecutively and are detailed explained in Tables S3.

**Figure 3:**
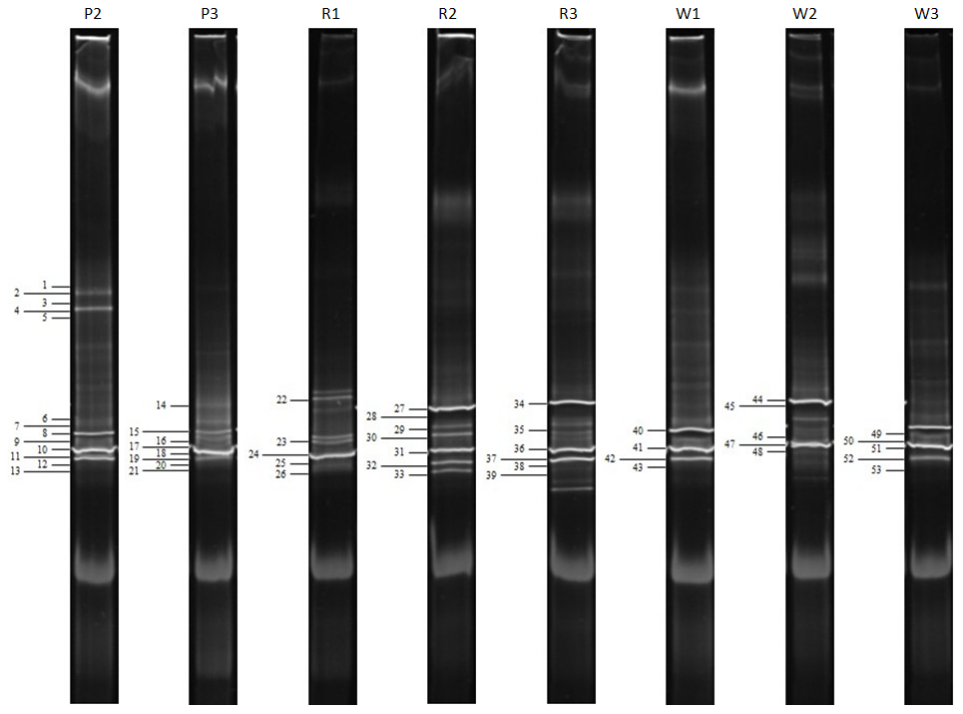
DGGE analysis of the archaeal communities present in the samples from the three buildings. The selected clone sequences are marked with bars, numbered consecutively and are detailed explained in Table S4.

The second part of each mixed sample was subjected to direct DNA extraction to further elucidate the non-cultivable microbiota. The bacterial 16S rDNA could be amplified by PCR using universal primers from all investigated samples. However, the amplification of archaeal ribosomal DNA was possible for nearly all samples except from sample P1 of Pürgg. DGGE fingerprint analyses were conducted with the amplified 16S rDNA to obtain information on the diversity present on the walls. The received fingerprints of the inhabiting bacterial and archaeal communities of all investigated samples from the three buildings are shown in Figures 2 and 3, respectively. The band numbers of all identified clones are indicated in Tables S3 and S4 and in Figures 2 and 3 to allow an easy tracking of the corresponding band in the community profile of each sample.

The bacterial fingerprints showed to be rather complex with three to seven dominant bands and many faint bands (Figure 2). The DGGE profiles from the different samples of each building and among the different buildings were very similar. The archaeal DGGE analysis showed rather simple banding profiles with two to four dominant bands and only a few faint bands (Figure 3). As already observed on the DGGE profiles derived from the bacteria, generally, the archaeal DGGE fingerprints showed a high homology among the different samples investigated from all three buildings.

### Phylogenetic identification of the bacterial clone sequences

A total of 107 clones were selected based on the DGGE patterns shown in Figure 2. Clones were sequenced and identified as unclassified Bacterium clone sequences (28% of all selected clones) and grouped to representatives of the following three phyla: Actinobacteria (67.3%), Firmicutes (2.8%) and Proteobacteria (1.9%). Generally, the bacterial 16S rDNA sequences from clone inserts showed similarities ranging from 92 to 100% to known sequences in the used databases (Table S3).

Generally, most identified 16S rDNA clone sequences affiliated with uncultured cloned sequences (75.7% of all selected clones) but also with some cultured bacterial strains (24.3%). Three cloned sequences, all belonging to the order Bacillales of the Firmicutes, were identified as the species *Natribacillus halophilus*, *Bacillus agaradhaerens* and as a *Planococcus* sp. Only two 16S rDNA sequences were related to members of the classes Beta- and Gammaproteobacteria, namely to *Ralstonia insidiosa* and an uncultured gammaproteobacterium. The majority of the detected clone sequences were affiliated with members of the phylum Actinobacteria. Thereof, 44 clones were related to the subclass Rubrobacteridae, six clones to the suborder Pseudonocardineae, two clones to the Micrococcineae and one clone each to Nocardioidaceae and Jiangellaceae, respectively. In Table 1 the distribution to the different phyla and genera is given for the bacterial clone sequences obtained from the samples of all three historical buildings.

### Phylogenetic identification of the non-cultivable archaeal community

The DGGE patterns of the archaeal community are shown in Figure 3. A total of 53 clones were chosen for sequence analysis. The results allowed a grouping into unclassified archaeon clones (26.4% of all selected clones) and representatives of the phylum Euryarchaeota, namely to the order Halobacteriales (73.6%). Thereof, 38 were grouped to the family Halobacteriaceae (71.7%) and further to the genera *Halococcus* (39.6%), *Halobacterium* (17%) and *Halalkalicoccus* (13.2%). In general, the comparative sequence analysis revealed similarity values ranging from 95 to 99% with known sequences from the NCBI database (Table S4).

As already observed for the bacterial cloned sequences, the identified archaeal 16S rDNA inserts generally affiliated with uncultured cloned sequences (62.3% of all selected clones) and to 20 cultured archaeal species (37.7%) in the database. Table 1 shows the distribution of the cloned archaeal 16S rDNA sequences to the different phyla and genera.

### Biodeterioration of building materials by halophilic microorganisms under laboratory conditions

**Figure 4:**
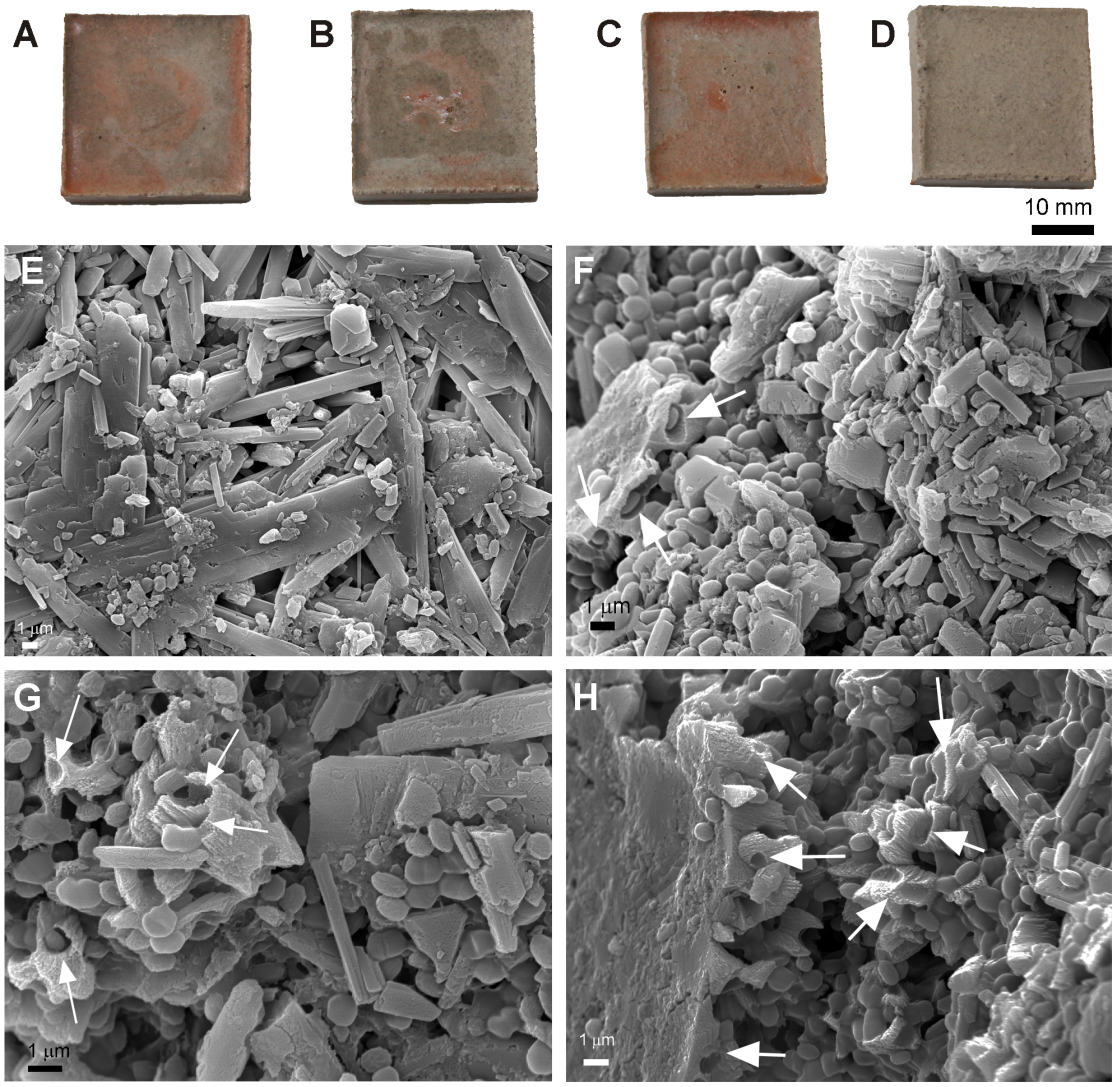
Colonization on gypsum plaster and FESEM images. Upper view of one representative gypsum plaster probe after one month of incubation: A) probe inoculated with *Halobacillus naozhouensis*, B) probe inoculated with *Kocuria polaris*, C) probe inoculated with the mixture of both strains, and D) non-inoculated gypsum plaster probe; and corresponding FESEM images depicting: E) gypsum mineral grains of a non-inoculated probe, F) microbial cells spread all over the surface and coccoid cells actively penetrating into the gypsum substratum (arrows), G) Gypsum minerals extensively etched (arrows, H) dissolution cavities or imprints of bacterial cells on calcium sulfate mineral grains. All photographs were taken by Cesareo Saiz-Jimenez and Ana Z. Miller, respectively.

**Figure 5:**
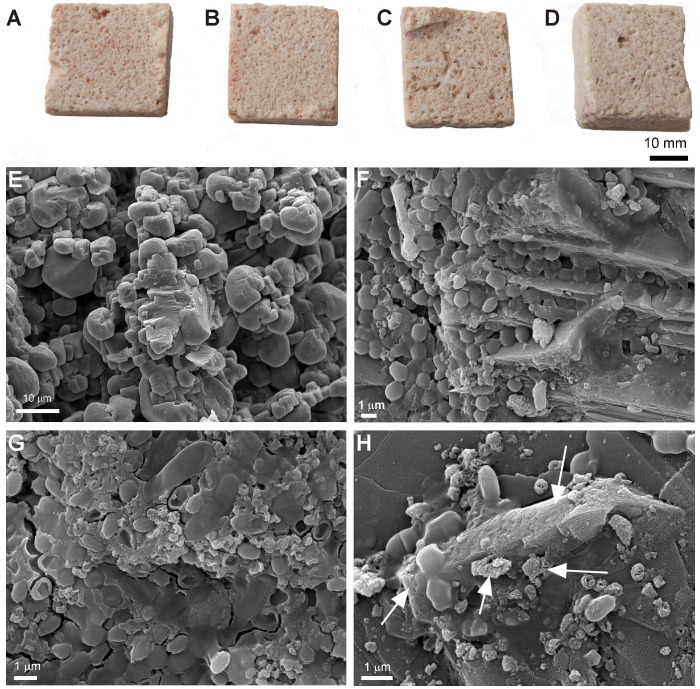
Colonization on limestone and FESEM images. Upper view of one representative Hontoria limestone probe after one month of incubation: A) probe inoculated with *Halobacillus naozhouensis*, B) probe inoculated with *Kocuria polaris*, C) probe inoculated with the mixture of both strains, and D) non-inoculated limestone probe; and FESEM images of the inoculated limestone probes depicting: E) calcium carbonate grains of a non-inoculated stone probe, F) microbial cells spread all over the surface and within stone cavities embedded in EPS, G) coccoid and rod-shaped cells on a limestone probe inoculated with both strains, H) calcium carbonate mineral grain showing signs of dissolution (arrows). All photographs were taken by Cesareo Saiz-Jimenez and Ana Z. Miller, respectively.

Visual inspection of the gypsum plaster and Hontoria limestone probes inoculated with Halobacillus naozhouensis and Kocuria polaris revealed that the laboratory-based colonization experiment led to the development of rosy coatings over the surface of the probes, particularly on the gypsum plaster (Figure 4A–D). Although less apparent, rosy discolouration was also observed on the limestone probes mainly within the pores of this lithotype due to its high open porosity and cell penetration into the limestone pores (Figure 5A–D).

FESEM images of the surface of the inoculated gypsum plaster and Hontoria limestone probes displayed the crystal morphology of gypsum and calcite minerals, respectively, as well as dense microbial mats spread all over the probe surfaces (Figure 4 and Figure 5). Great amount of coccoid cells, corresponding to Kocuria polaris, embedded in extracellular polymeric substances (EPS) were found on both type of materials (Figure 4F, G and Figure 5F, G). Rod-shaped cells were less frequently observed on the probes inoculated with both strains (Figure 5G).

In addition, gypsum mineral grains were found extensively etched, evidencing microbe-mineral interactions. The etched mineral surfaces presented imprints of microbial cells, rough texture and rounded edges (Figure 4F, G). Active dissolution is evidenced by the penetration of coccoid cells into the mineral substratum, producing etching features in the form of shallow imprints or cavities on the mineral substratum (Figure 4F, G, H arrows). In Figures 4G and 4H it is also clearly noticeable the signs of dissolution on the crystal surfaces, which were not observed on the non-inoculated gypsum plaster probes (Figure 4E). These dissolution features were much lesser noticed on the surface of calcite crystals than on the gypsum substratum (Figure 5F, H arrows).

## Discussion

In this study culture-based and molecular methods were combined to get a complete overview of the microbial communities responsible for the rosy pigmentation in the buildings. The classical cultivation techniques offer the possibility to isolate the microorganisms responsible of the phenomenon and to reproduce it in the laboratory. Moreover, cultivation offers the ability to visualize the pigmentation of the grown strains as an important agent of aesthetical damage on the objects. Different culture media that were already successfully used for the isolation of microorganisms from historical buildings [18] were applied. Additionally, the composition of the used media was adapted to the natural conditions by adding different salts in various concentrations. Nevertheless, it is well-known that by using only standard cultivation techniques with conventional laboratory media, only a small proportion of the total inhabiting bacterial population can be cultivated [32]. Therefore, molecular techniques using PCR amplification of ribosomal 16S RNA genes, creation of clone libraries and the screening by DGGE and identification via sequence analysis, were additionally used in this project. This combination of different microbiological methods allowed the coverage of a wider spectrum of the microbial ecosystem present in the rosy discoloured wall materials.

Compared to the culture dependent approach, molecular analysis showed a higher biodiversity, with bacteria belonging to different genera. Generally, 57.5% of all 16S rDNA sequences were originally detected in historical buildings or paintings from which 62.6% corresponded to bacterial- and 47.2% to archaeal clone sequences (Table 2). Also 55.2% of the bacterial isolates were previously found in this type of buildings. The bacterial clone sequences showed a great dominance of members of the Actinobacteria, where more than 67% of all analysed sequences affiliated with this phylum (Table 1). Thereof, 41.1% grouped to the subclass Rubrobacteridae. About 9 to 19% of all identified clone sequences were also detected on one or both of the other buildings investigated in this study (subsets in Table 2).

**Table 2:** Sequence affiliations.

16S rDNA clones											
Bacteria	Affiliation with sequence from				Clones without a match			Clones matching each other from different buildings			
	historical building/painting	saltenvironment	soil	otherenvironments	P	R	W	P⊆R	P⊆W	R⊆W	P⊆R⊆W
**P-clones** (41)	25 (61%)	3 (7.3%)	12 (29.3%)	1 (2.4%)	13 (31.7%)			10 (24.4%)	9 (22%)		9 (22%)
**R-clones** (35)	20 (57.1%)	6 (17.1%)	9 (25.7%)			20 (57.1%)		5 (14.3%)		5 (14.3%)	5 (14.3%)
**W-clones** (31)	22 (71%)	2 (6.5%)	6 (19.4%)	1 (3.2%)			9 (29%)		11 (35.5%)	5 (16.1%)	6 (19.4%)
***Total no.*** * (107)*	*67 (62.6%)*	*11 (10.3%)*	*27 (25.2%)*	*2 (1.9%)*	13 (12.1%)	20 (18.7%)	9 (8.4%)	15 (14%)	20 (18.7%)	10 (9.3%)	20 (18.7%)

Archaea	Affiliation with sequence from				Clones without a match			Clones matching each other from different buildings			
	historical building/painting	saltenvironment	soil	otherenvironment	P	R	W	P⊆R	P⊆W	R⊆W	P⊆R⊆W
**P-clones** (21)	11 (52.4%)	5 (23.8%)	4 (19%)	1 (4.8%)	10 (47.6%)			3 (14.3%)	6 (28.6%)		2 (9.5%)
**R-clones** (18)	6 (33.3%)	9 (50%)	1 (5.6%)	2 (11.1%)		9 (50%)		3 (16.7%)		4 (22.2%)	2 (11.1%)
**W-clones** (14)	8 (57.1%)	4 (28.6%)		2 (14.3%)			2 (14.3%)		5 (35.7%)	3 (21.4%)	4 (28.6%)
***Total no.*** * (53)*	*25 (47.2%)*	*18 (34%)*	*5 (9.4%)*	*5 (9.4%)*	10 (18.9%)	9 (17%)	2 (3.8%)	6 (11.3%)	11 (20.8%)	7 (13.2%)	8 (15.1%)
**Bacterial isolates**											

	Affiliation with sequence from				Clones without a match		Clones matching each other from different buildings				
	historical building/painting	saltenvironment	soil	otherenvironments	P	R	P⊆R				
**P-samples** (12)	10 (83.3%)	2 (16.7%)			2 (16.7%)		10 (83.3%)				
**R-samples** (17)	6 (35.3%)	6 (35.3%)	3 (17.6%)	2 (11.8%)		12 (70.6%)	5 (29.4%)				
***Total no.*** * (29)*	*16 (55.2%)*	*8 (27.6%)*	*3 (10.3%)*	*2 (6.9%)*	2 (6.9%)	12 (41.4%)	15 (51.7%)				

Overview of the 16S rDNA sequences from the bacterial- and archaeal clones sequences as well as the bacterial isolates from this study that affiliated with known sequences from the NCBI database, which were previously detected from different environments. Number of clones and calculated percentages indicate the relatedness to these habitats as well as the subsets of the sequences derived from different buildings to each other.

Forty-four sequences (27.5%) were related to samples taken from the Saint Catherine chapel in castle Herberstein (Austria). The walls of this chapel also showed a strong rosy discolouration due to microbial colonization. Nineteen sequences (11.9%) were related to sequences retrieved from different studies performed at the subterranean Saint Virgil chapel in Vienna. The subterranean chapel suffered from water-infiltrations through the walls and as a result widespread salt efflorescences were visible by naked eye in the whole building [10]–[15], [17], [33].

Fifteen sequences (9.4%) were related to sequences detected in a study performed by Piñar et al. [16] about the microbiota in the Capuchin catacombs of Palermo (Italy). Also there - and similar to the Saint Virgil chapel - water was migrating horizontally into the walls, thus leading to extensive rosy discolourations and the precipitation of salts on the walls. Another fifteen sequences (9.4%) showed to be related with cultured strains isolated from rosy discoloured ancient wall paintings of the Crypt of the Original Sin (Matera, Italy) which was performed by Imperi et al. [3].

Jurado et al. [18] and Laiz et al. [34] identified a *Rubrobacter* community in Roman tombs (Carmona, Spain) and the interior walls of the Vilar de Frades Church (Barcelos, Portugal). In this study seven (4.4%) 16S rDNA sequences that were related to the mentioned works were found. Finally eight sequences (5%) were related to four different historical locations, which partly also showed the pink pigmentation phenomenon: the church of Saint Anna im Feld, Germany [17], the Roman Necropolis of Carmona, Spain [14], [29], [34], the Tomb of the Monkeys, Italy [35] and an old mould-damaged building [36].

Members of the genus *Rubrobacter* represented around 20–26% of clones retrieved from the three monuments studied. The species of this genus are difficult to isolate, required culture media specifically designed for such purpose and some of the strains obtained represented new species [18]. A novel *Rubrobacter* species isolated from Roman tombs [34] is waiting for description. *Rubrobacter* seems to be associated to phototrophic microorganisms as illustrate Figure 1C and reported by Laiz et al. [34]. These authors stated that *Rubrobacter* strains play an active role in efflorescence niches and in mineral precipitation, and contribute to biodeterioration processes.

Classical cultivation experiments allowed the isolation of *Halomonas muralis* which was previously cultivated by Heyrman et al. [33] from the walls of the Saint Catherine chapel from castle Herberstein. Four years later Ripka et al. [13] were able to cultivate different *Halobacillus* species (*Halobacillus herbersteinensis*) from the same location. We were able to isolate a similar strain from samples of the Johannes chapel in Pürgg as well as from the castle Rappottenstein, showing that 51.7% of all isolates were detected on both buildings (subsets in Table 2).

Further eight isolates (27.6%) were originally found in saline environments, like marine solar salterns (*Planococcus salinarum* and *Planomicrobium flavidum*) [37], [38], marine sediments (*Kocuria rosea*) [39] or a salt mine (*Marinococcus luteus*) [40], respectively. *Planococcus donghaensis*, *Paracoccus marcusii* and *Planococcus antarcticus* were previously detected in soil material from the Antarctic, from coastal regions in South Korea [41] and in soil of Issyk Kul region in Kyrgyzstan, respectively.

Bacterial strains that showed a yellow, orange or pink colour when cultivated on different media were selected for this study. Additionally, some of the microorganisms that were detected with molecular methods are also known for their pigmented colonies ranging from light yellow to light pink, orange, rosy to red or brown due to the production of the characteristic carotenoids bacterioruberin and monoanhydrobacterioruberin such as *Nesterenkonia xinjiangensis*, *Janibacter corallicola*, *Natribacillus halophilus*, *Saccharopolyspora salina* and *Rubrobacter* sp. [18], [34], [42], [43]. All of those strains are moderately to extremely halophilic bacteria that can grow up to a maximum NaCl concentration of 7–23% (w/v).

By using culture dependent techniques members of the Firmicutes phylum were predominantly found in the samples (89.7%), whereas the actinobacterial fraction represented the smallest part of the isolated strains (3.5%). Conversely, the application of molecular methods for the identification of the bacteria showed primarily representatives of the Actinobacteria (67.3%) and the Firmicutes accounted only for 2.8% of the cloned sequences.

In a previous study 47.1% of the isolated bacteria from the Saint Virgil Chapel belonged to the *Halobacillus* genus [10]. In this study even a higher percentage of the cultured strains (55.2%) were affiliated with this genus. However, it is worth noting that the culture-independent analysis of the samples did not yield any clones harbouring halobacilli sequences. A similar pitfall of molecular analysis was already observed by Piñar et al. [12] during the observation of the microbiota in the Saint Virgil chapel and were discussed by several authors [24], [44]–[49]. The disparities in the results obtained by culture dependent and –independent techniques in this study once more show the drawbacks of each approach for an accurate description of the microbial community in a certain habitat [50], [51].

We could again proof the co-existence of moderately halophilic bacteria and neutrophilic halophilic archaea on hypersaline environments represented by historical stone works, which was already shown before [10], [12], [52]. Similar to the bacterial clone sequences, the majority of the archaeal 16S rDNA sequences showed to be related to mural stonework or ancient paintings (47.2%; Table 2). The identified archaeal sequences were previously detected either in the Saint Virgil chapel, the Saint Catherine chapel or the Capuchin catacombs. Thirty-four percent of all analyzed archaeal clones were originally found in saline environments, e.g. in a solar saltern in Greece (*Halococcus* sp.) [53] or in different saline sediments (*Halalkalicoccus* sp., Haloarchaeon Nie 13) [54], [55]. A further 18.8% have diverse origins (soil, groundwater, etc.).

Some of the identified archaeal species also produce colourful pigments as means of protection against exposure to UV light and chemicals. *Halalkalicoccus* sp. and *Natronorubrum* sp. show a pink-pigmented colour appearance [56], [57], whereas bright orange to pink colonies are formed by *Halococcus hamelinensis* [58]. These haloarchaea are able to grow on even higher salt concentrations than the detected halophilic bacteria – up to 30% NaCl (w/v) [59].

The interaction between microorganisms and mineral substrata was studied by FESEM in order to address the real action of *Halobacillus naozhouensis* and *Kocuria polaris* on gypsum plaster and limestone probes, and certain biodeterioration phenomenon on these building materials. The distribution pattern of the rosy biofilms on the inoculated probes was different for both materials due to their petrophysic characteristics, mainly, stone surface roughness. The presence of larger pores on the Hontoria limestone allowed the development of the inoculated microorganisms within the stone probes, contrasting with the smoother surface of the gypsum plaster. The development and activity of these microorganisms on both substrata were responsible for the rosy discolouration and might also cause the dissolution features observed by FESEM, particularly on the gypsum mineral grains. Biogeochemical deterioration is the direct action caused by the metabolic processes of organisms on a substratum [60]. The biogenic release of corrosive acids is probably the best well-known and most commonly investigated biogeochemical damage mechanism in inorganic materials. The process known as biocorrosion, involves the release of organic acids which can etch or solubilize stone minerals [61]. The dissolution features observed on the inoculated gypsum plaster probes is a clear evidence of the microbial activity present on these mineral substrata inducing biodeterioration. Microorganisms may also induce biodeterioration though actively dissolution of carbonates and other minerals to enable penetration into the substratum enhancing stone porosity [62], [63]. These biogeochemical processes give rise to changes on the lithic substratum, as particularly observed on the inoculated gypsum plaster probes (Figure 4). The data shows that the rosy discolouration phenomenon, in addition to an unaesthetic effect, induces also biogeochemical deterioration.

## Conclusions

In this work we could show that the rosy biofilms on the walls of three different buildings harbour very similar bacterial and archaeal communities. Similar climatic conditions with relatively low UV irradiations and lowered annual temperatures, constructional problems with water infiltrations into the walls, the migration and further crystallisation of salts on the surface lead to the formation of extreme saline environments that offer optimal growth conditions for halophilic microorganisms. The inhabiting members of the Firmicutes and Actinobacteria, mainly representatives of the subclass Rubrobacteridae, as well as Halobacteriales members are the main cause for the rosy coloured biofilms on the walls. These microorganisms were already detected in other historical buildings from different locations in Europe. Further investigations should address their goals in the design of special cultivation media to isolate the so far unidentified members of the *Rubrobacter* genus and the Halobacteriales order, which were also involved in this phenomenon.

The results of this study show that halotolerant and halophilic microbes with brilliant rosy to purple colourations are the most important biodeteriogens of walls and wall paintings in salt-burdened historical sites. The intensity of the stains often is a serious aesthetical damage of wall paintings and in some cases it might even lead to an illegibility of the painting. For this reason, restorers often wish to carry out a treatment to remove this microbiota from the surfaces. Since desalination – by use of compresses – is a general tool in order to decrease the salt crystallization and the mechanical damage related to this, this method could also help to stop the growth of halophilic and halotolerant microorganisms. However, without any accompanying measures that decrease the humidity, the habitat would then be open for a wide variety of less salt-tolerant microbes including fungi [12]. Since especially fungi are very potent producers of organic acids and also decomposers of organic binders in wall paintings, such a microbiota might be even more harmful for the object than the predominantly aesthetical damage caused by pigmented bacteria and archaea. Therefore, a desalination of the walls is only reasonable in combination with structural measures that decrease the humidity down to a level that does not allow microbial growth. Such measures could be drainage and repair of constructional damages or better ventilation to avoid condensation. The same holds true for the application of biocides. None of the biocide compounds that are currently used in restoration – including quaternary ammonium compounds, ethanol or formaldehyde-releasers – have a preventive effect against re-colonization. Thus, application of a biocide can only be recommended in parallel to climate control measures. If climate control is impossible, it should be considered to accept the coloured microbiota rather than disturbing or changing the microbial community by treatments like desalination or application of biocides that, if not ineffective, can cause more fatal damage to the paintings [5], [64].

## Supporting Information

Figure S1 **Additional information about the buildings.** The Johannes Chapel in Pürgg (A, B), the castle Rappottenstein (C, D) and the Saint Rupert Chapel in Weißpriach (E, F). A) The Johannes chapel in Pürgg was first restored between 1889 and 1894 in the sense of historicism, which was again removed between 1939 and 1949 where all original paintings were restored. B) The chapel is built on top of a hill and due to the strong exposure to the weather, in the 1960s a shingles wall was installed outside of the west wall to protect it against rain, wind and snow. Since 1996 a few additional actions to protect the chapel were made: The whole chapel received an outside exterior rendering with lime plaster, the entrance was moved from the west side to the north with an additional small room as climatic sluice. These constructional changes led to a cooling of the west wall and the further establishment of a rosy colour on the whole wall. C) The castle Rappottenstein was built on a hill and therefore under strong atmospheric exposure. The walls in the inner courtyard are highly exposed to rain and snow as well as water migrates through the walls of the whole building leading to the formation of salt efflorescences. The castle contains arcaded sidewalks with Sgraffiti decoration over three floors and famous frescos with profane-paintings from the 16th century. D) The famous “Green room”, also called “Brudermordzimmer” (brother-murderer-room), on the 2nd floor shows medieval scenes on green background and dates back to 1520-1480. E) Only 50 kilometres away from Pürgg is the Saint Rupert chapel of Weißpriach that, due to its geographical location in the Alps, is also exposed to alpine climatic conditions. The chapel consists of a north orientated tower and an inner hall with rectangular choir with a semicircle apse. F) The most outstanding murals were discovered in 1949 and also in 1977/78. The “Last Judgment” in two registers, the “Legend of Ägidius”, hunting scenes of Visigoth kings and martyr were found underneath the plaster, laid open and subsequent restored. All photographs were taken by Jörg D. Ettenauer.
(TIF)

Table S1 **Description of the pooled and further analysed samples from the three historical buildings.** The compositions of the mixed samples with the original samples numbers, sample amounts (in gram) as well as the mixed sample amounts (in gram) are given that were further used for cultivation- and molecular analysis.
(DOCX)

Table S2 **Phylogenetic affiliations of isolated strains.** Phylogenetic affiliations of the 16S rRNA gene sequences obtained from the cultivated bacteria in the samples from Pürgg (P2) and Rappottenstein (R1, R2 and R3). The number of isolates, the colour appearance, the growth conditions and isolation time after incubation start (in hours and days), the sequence length of the 16Sr DNA for database comparison, the similarity of the closest relative from the NCBI- and EZtaxon- (marked with an asterisk*) database and the accession numbers are given. Accession codes: Sequences were deposited at the NCBI GenBank under the accession numbers HG515390–HG515401.
(DOCX)

Table S3 **Phylogenetic affiliations of the bacterial sequences.** Phylogenetic affiliations of the partial 16S rRNA gene sequences obtained from all bacterial clones of the samples from the three buildings. Accession codes: Sequences were deposited at the NCBI GenBank under the accession numbers KF692550–KF692709 for the cloned sequences.
(DOCX)

Table S4 **Phylogenetic affiliations of the archaeal sequences.** Phylogenetic affiliations of the partial 16S rRNA gene sequences obtained from all archaeal clones in the samples from the three buildings. Accession codes: Sequences were deposited at the NCBI GenBank under the accession numbers KF692550–KF692709 for the cloned sequences.
(DOCX)
