# Supplementary figures and images for: Halophilic Microorganisms Are Responsible for the Rosy Discolouration of Saline Environments in Three Historical Buildings with Mural Paintings
Source: PLoS One. 2014 Aug 1;9(8):e103844. doi: 10.1371/journal.pone.0103844 (PMC4118916; doi:10.1371/journal.pone.0103844)

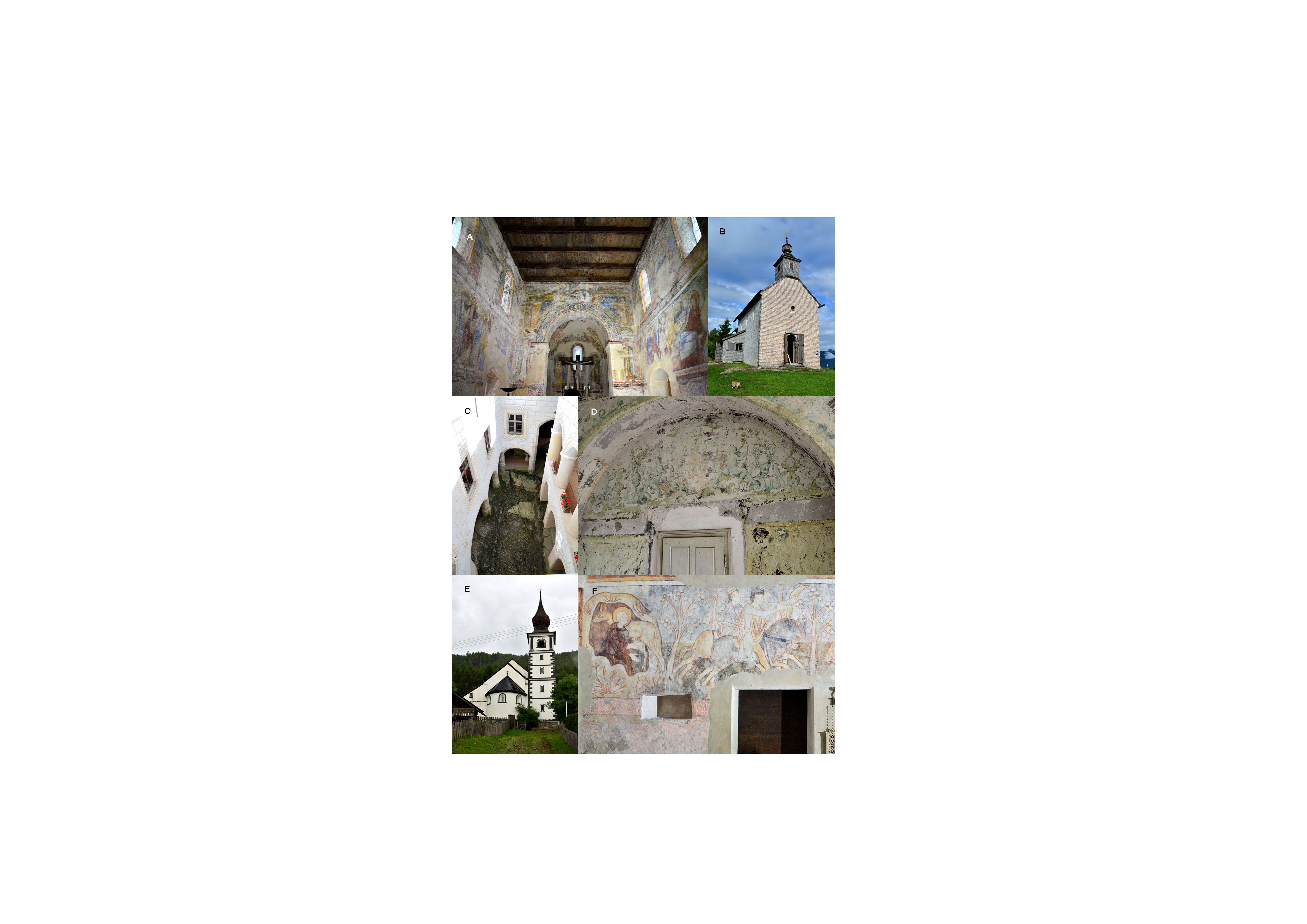

Supplement: Figure S1 — Additional information about the buildings. The Johannes Chapel in Pürgg (A, B), the castle Rappottenstein (C, D) and the Saint Rupert Chapel in Weißpriach (E, F). A) The Johannes chapel in Pürgg was first restored between 1889 and 1894 in the sense of historicism, which was again removed between 1939 and 1949 where all original paintings were restored. B) The chapel is built on top of a hill and due to the strong exposure to the weather, in the 1960s a shingles wall was installed outside of the west wall to protect it against rain, wind and snow. Since 1996 a few additional actions to protect the chapel were made: The whole chapel received an outside exterior rendering with lime plaster, the entrance was moved from the west side to the north with an additional small room as climatic sluice. These constructional changes led to a cooling of the west wall and the further establishment of a rosy colour on the whole wall. C) The castle Rappottenstein was built on a hill and therefore under strong atmospheric exposure. The walls in the inner courtyard are highly exposed to rain and snow as well as water migrates through the walls of the whole building leading to the formation of salt efflorescences. The castle contains arcaded sidewalks with Sgraffiti decoration over three floors and famous frescos with profane-paintings from the 16th century. D) The famous “Green room”, also called “Brudermordzimmer” (brother-murderer-room), on the 2nd floor shows medieval scenes on green background and dates back to 1520-1480. E) Only 50 kilometres away from Pürgg is the Saint Rupert chapel of Weißpriach that, due to its geographical location in the Alps, is also exposed to alpine climatic conditions. The chapel consists of a north orientated tower and an inner hall with rectangular choir with a semicircle apse. F) The most outstanding murals were discovered in 1949 and also in 1977/78. The “Last Judgment” in two registers, the “Legend of Ägidius”, hunting scenes of Visigoth kings and [file pone.0103844.s001.tif]
