# Supplementary material for: Halophilic Microorganisms Are Responsible for the Rosy Discolouration of Saline Environments in Three Historical Buildings with Mural Paintings
Source: PLoS One. 2014 Aug 1;9(8):e103844. doi: 10.1371/journal.pone.0103844 (PMC4118916; doi:10.1371/journal.pone.0103844)
Supplement: Table S1 — Description of the pooled and further analysed samples from the three historical buildings. The compositions of the mixed samples with the original samples numbers, sample amounts (in gram) as well as the mixed sample amounts (in gram) are given that were further used for cultivation- and molecular analysis. (DOCX) [file pone.0103844.s002.docx]

**Supporting Table S1.** **Description of the pooled and further analysed samples from the three historical buildings.** The compositions of the mixed samples with the original samples numbers, sample amounts (in gram) as well as the mixed sample amounts (in gram) are given that were further used for cultivation- and molecular analysis.

| **Location** | **Mixed samples** | **Taken sample no.** | **Location** | **Sample amount** | **Mixed sample amount** |
| --- | --- | --- | --- | --- | --- |
| **Pürgg** | **P1** | 1 | chapel entry (north wall) | 3.39 | 5.16 |
|  |  | 2 | chapel entry (north wall) | 0.5 |  |
|  |  | 3 | chapel entry (north wall) | 0.7 |  |
|  |  | 4 | chapel entry (north wall) | 0.57 |  |
|  | **P2** | 5 | west wall | 1.42 | 16.86 |
|  |  | 6 | west wall | 0.92 |  |
|  |  | 7 | west wall | 14.52 |  |
|  | **P3** | 8 | east wall | 5.36 | 5.77 |
|  |  | 9 | east wall | 0.25 |  |
|  |  | 10 | east wall | 0.16 |  |
| **Weißpriach** | **W1** | 1 | tower-room | 1.8 | 3.37 |
|  |  | 2 | tower-room | 1.57 |  |
|  | **W2** | 3 | north wall | 0.76 | 0.76 |
|  | **W3** | 4 | south wall | 1.65 | 1.65 |
| **Rappottenstein** | **R1** | 1 | ground floor | 10.16 | 30.42 |
|  |  | 2 | ground floor | 0.23 |  |
|  |  | 3 | ground floor | 2.67 |  |
|  |  | 4 | ground floor | 0.3 |  |
|  |  | 5 | ground floor | 0.57 |  |
|  |  | 6 | ground floor | 0.91 |  |
|  |  | 7 | ground floor | 14.46 |  |
|  |  | 8 | ground floor | 1.12 |  |
|  | **R2** | 9 | second floor | 3.71 | 10.89 |
|  |  | 10 | second floor | 0.3 |  |
|  |  | 11 | second floor | 1.18 |  |
|  |  | 12 | second floor | 5.7 |  |
|  | **R3** | 13 | first floor | 8.82 | 11.08 |
|  |  | 14 | first floor | 0.93 |  |
|  |  | 15 | first floor | 0.49 |  |
|  |  | 16 | first floor | 0.84 |  |
