# Supplementary material for: Halophilic Microorganisms Are Responsible for the Rosy Discolouration of Saline Environments in Three Historical Buildings with Mural Paintings
Source: PLoS One. 2014 Aug 1;9(8):e103844. doi: 10.1371/journal.pone.0103844 (PMC4118916; doi:10.1371/journal.pone.0103844)
Supplement: Table S2 — Phylogenetic affiliations of isolated strains. Phylogenetic affiliations of the 16S rRNA gene sequences obtained from the cultivated bacteria in the samples from Pürgg (P2) and Rappottenstein (R1, R2 and R3). The number of isolates, the colour appearance, the growth conditions and isolation time after incubation start (in hours and days), the sequence length of the 16Sr DNA for database comparison, the similarity of the closest relative from the NCBI- and EZtaxon- (marked with an asterisk*) database and the accession numbers are given. Accession codes: Sequences were deposited at the NCBI GenBank under the accession numbers HG515390–HG515401. (DOCX) [file pone.0103844.s003.docx]

**Supporting Table S2.** **Phylogenetic affiliations of isolated strains.** Phylogenetic affiliations of the 16S rRNA gene sequences obtained from the cultivated bacteria in the samples from Pürgg (P2) and Rappottenstein (R1, R2 and R3). The number of isolates, the colour appearance, the growth conditions and isolation time after incubation start (in hours and days), the sequence length of the 16Sr DNA for database comparison, the similarity of the closest relative from the NCBI- and EZtaxon- (marked with an asterisk*) database and the accession numbers are given. Accession codes: Sequences were deposited at the NCBI GenBank under the accession numbers HG515390-HG515401.

| **Sample** | **No. of isolates** | **Colour-appearance** | **Culture medium Incubation time** | **Sequence length (bp)** | **Nearest published relative and isolation source from NCBI- and from EZtaxon* database** | **Similarity (%)** | **Accession No.** |
| --- | --- | --- | --- | --- | --- | --- | --- |
| **P2** | 2 | Orange | TSA-NaMg, 48h | 1441 | *Planococcus salinarum* strain ISL-16 [FJ765415.1] from a marine solar saltern | 98  98.6* | HG515390 |
|  | 10 | Lightly pink - pink | TSA-NaMg15%, 6d  M1018, 6d  M372, 12d | 1504 | *Halobacillus herbersteinensis* strain I7 [AM161504.1] from medieval paintings and stone-works in Castle of Herberstein, Styria, Austria *Halobacillus naozhouensis* JSM 071068(T) [EU925615.1] from a sea anemone* | 99  98.5* | HG515391 |
| **R1** | 1 | Orange | TSA-NaMg, 7d | 1382 | *Paracoccus marcusii* isolate SCH0403 [AY881236.1] from east and south coast, South Korea  *Paracoccus marcusii* DSM 11574(T) [Y12703.1] isolated as a contaminant on a nutrient agar plate* | 100  100* | HG515392 |
|  | 3 | Orange – Yellow  Lightly orange | TSA-NaMg15%, 7d  M1018, 7d | 1456 | *Marinococcus luteus* strain KCTC 13214 [AB769483.1] from a salt mine located in Tarija, Bolivia  *Marinococcus tarijensis* SR-1(T) [JQ413413.1] from a salt mine located in Tarija, Bolivia* | 99  99.7* | HG515394 |
|  | 5 | Pink | M1018, 7d  TSA-NaMg15%, 7d | 1468 | *Halobacillus herbersteinensis* strain I7 [AM161504.1] from medieval paintings and stone-works in Castle of Herberstein, Styria, Austria  *Halobacillus naozhouensis* JSM 071068(T) [EU925615.1] from a sea anemone* | 99  99.3* | HG515393 |
|  | 1 | Lightly orange | TSA-NaMg15%, 8d | 1436 | *Halomonas muralis* strain LMG 20969 [NR_025486.1] from microbial biofilms on walls and murals in the Saint-Catherine chapel, Castle Herberstein, Austria | 98  97.8* | HG515395 |
| **R2** | 1 | Orange | TSA-NaMg, 48h | 1436 | *Planococcus psychrotoleratus* [AY771711.1]  *Planomicrobium okeanokoites* IFO 12536(T) [D55729.1] from a marine mud* | 99  98.5* | HG515396 |
|  | 2 | Orange | Marine Agar, 48h, Marine Agar, 5d | 1418 | *Planomicrobium flavidum* strain ISL-41 [FJ265708.1] from a marine solar saltern | 98  98.30* | HG515398 |
|  | 1 | Orange | Marine Agar, 48h | 1439 | *Planococcus donghaensis* strain L10.15 [KC479344.1] from soil in the Antarctic  *Planococcus donghaensis* JH 1(T) [EF079063.1] from deep-sea sediment of the East Sea, South Korea* | 99  99.1* | HG515397 |
|  | 1 | Pink - Orange | TSA-NaMg, 5d | 1479 | *Kocuria rosea* [DQ060382.1] from the Arctic Ocean marine sediments  *Kocuria polaris* CMS 76or(T) [AJ278868.1] from an Antarctic cyanobacterial mat sample* | 99  99.5* | HG515399 |
|  | 1 | Orange | Marine Agar, 7d | 1440 | *Planococcus antarcticus* strain B-9 [KF318398.1] from soil of Issyk Kul region, Kyrgyzstan  *Planococcus donghaensis* JH 1(T) [EF079063.1] from deep-sea sediment of the East Sea, South Korea* | 99  98.7* | HG515400 |
| **R3** | 1 | Orange | Marine Agar, 48h | 1440 | *Planococcus psychrotoleratus* [AF324659.1]  *Planococcus donghaensis* JH 1(T) [EF079063.1] from deep-sea sediment of the East Sea, South Korea* | 99  98.9* | HG515401 |
