# Supplementary material for: Halophilic Microorganisms Are Responsible for the Rosy Discolouration of Saline Environments in Three Historical Buildings with Mural Paintings
Source: PLoS One. 2014 Aug 1;9(8):e103844. doi: 10.1371/journal.pone.0103844 (PMC4118916; doi:10.1371/journal.pone.0103844)
Supplement: Table S3 — Phylogenetic affiliations of the bacterial sequences. Phylogenetic affiliations of the partial 16S rRNA gene sequences obtained from all bacterial clones of the samples from the three buildings. Accession codes: Sequences were deposited at the NCBI GenBank under the accession numbers KF692550–KF692709 for the cloned sequences. (DOCX) [file pone.0103844.s004.docx]

**Supporting Table S3.** **Phylogenetic affiliations of the bacterial sequences.** Phylogenetic affiliations of the partial 16S rRNA gene sequences obtained from all bacterial clones of the samples from the three buildings. Accession codes: Sequences were deposited at the NCBI GenBank under the accession numbers KF692550-KF692709 for the cloned sequences.

| **DGGE band** | **Clone number** | **Sequence length [bp]** | **Nearest published relative and isolation source from NCBI database** | **Similarity**  **(%)** | **Accession number** |
| --- | --- | --- | --- | --- | --- |
| **1** | P1-57 | 396 | Uncultured *Rubrobacter* sp., clone K7 [AM161166.1] from mural paintings in the Chapel of St. Virgil, Vienna, Austria | 100 | KF692659 |
| **2** | P1-81 | 588 | *Rubrobacter* sp. CBF L56 [AB166956.1] from deep-sea sediment, Central Basin Fault, West Philippine Basin | 98 | KF692660 |
| **3** | P1-75 | 588 | Uncultured actinobacterium*,* clone E6 [KC442786.1] from soils of the Lake Wellman Area, Darwin Mountains, South Victoria Land, Antarctica | 99 | KF692704 |
| **4** | P1-47 | 588 | Uncultured eubacterium Hb7-K2 [AJ298575.1] from rosy discolouration of masonry and lime wall paintings | 99 | KF692661 |
| **5** | P1-82 | 588 | Uncultured eubacterium Hb7-K1 [AJ298576.1] from rosy discolouration of masonry and lime wall paintings | 99 | KF692662 |
| **6** | P1-37 | 588 | Uncultured *Rubrobacter* sp., clone B8-K12 [KC535155.1] from wall with purple stains in the Capuchin catacombs in Palermo, Italy | 99 | KF692663 |
| **7** | P1-29 | 588 | Uncultured actinobacterium*,* clone F15cmFL474 [JN002715.1] from serpentinized dunite of ultramafic rocks in the Leka ophiolite complex | 99 | KF692703 |
| **8** | P1-83 | 588 | *Rubrobacter* sp. VF70612_S1 [EU512991.1] from biodeteriorated monuments, Vilar de Frades Church, Barcelos, Portugal | 99 | KF692664 |
| **9** | P1-77 | 588 | Uncultured actinobacterium*,* clone F15cmFL453 [JN002699.1] from serpentinized dunite of ultramafic rocks in the Leka ophiolite complex | 99 | KF692705 |
| **10** | P1-96 | 588 | Uncultured bacterium, clone A6 [AM746684.1] from rosy-discoloured mural wall painting of the Crypt of the Original Sin, Matera, Italy | 99 | KF692665 |
| **11** | P1-46 | 588 | Uncultured bacterium, clone A6 [AM746684.1] from rosy-discoloured mural wall painting of the Crypt of the Original Sin, Matera, Italy | 99 | KF692666 |
| **12** | P1-68 | 587 | Uncultured eubacterium Hb7-K2 [AJ298575.1] from rosy discolouration of masonry and lime wall paintings | 97 | KF692667 |
| **13** | P1-17 | 588 | Uncultured eubacterium Hb7-K2 [AJ298575.1] from rosy discolouration of masonry and lime wall paintings | 99 | KF692611 |
| **14** | P2-42 | 504 | Uncultured *Rubrobacter* sp., clone K8 [AM161167.1] from mural paintings in the Chapel of St. Virgil, Vienna, Austria | 99 | KF692612 |
| **15** | P2-14 | 572 | *Nesterenkonia xinjiangensis,* strain YIM70097 [NR_029075.1] from saline soils in the west of China | 94 | KF692613 |
| **16** | P2-71 | 588 | Uncultured eubacterium MP1-K4 [AJ298571.1] from rosy discolouration of masonry and lime wall paintings | 99 | KF692614 |
| **17** | P2-34 | 588 | Uncultured eubacterium Hb7-K2 [AJ298575.1] from rosy discolouration of masonry and lime wall paintings | 99 | KF692615 |
| **18** | P2-15 | 588 | Uncultured eubacterium Hb7-K1 [AJ298576.1] from rosy discolouration of masonry and lime wall paintings | 99 | KF692616 |
| **19** | P2-9 | 588 | Uncultured *Rubrobacter* sp., clone B8-K12 [KC535155.1] from wall with purple stains in the Capuchin catacombs in Palermo, Italy | 99 | KF692617 |
| **20** | P2-91 | 566 | *Amycolatopsis* sp. 2-5 [GU132436.1] from Nalaikh coal mining site soil, Mongolia | 97 | KF692618 |
| **21** | P2-18 | 590 | Uncultured eubacterium Hb7-K1 [AJ298576.1] from rosy discolouration of masonry and lime wall paintings | 99 | KF692619 |
| **22** | P2-90 | 588 | Uncultured actinobacterium*,* clone F15cmL146 [JN002733.1] from serpentinized dunite of ultramafic rocks in the Leka ophiolite complex | 99 | KF692706 |
| **23** | P2-1 | 589 | Uncultured bacterium, clone A7 [AM746685.1] from rosy-discoloured mural wall painting of the Crypt of the Original Sin, Matera, Italy | 95 | KF692620 |
| **24** | P2-61 | 588 | Uncultured bacterium, clone A6 [AM746684.1] from rosy-discoloured mural wall painting of the Crypt of the Original Sin, Matera, Italy | 99 | KF692621 |
| **25** | P2-22 | 530 | Uncultured bacterium clone RamatNadiv03b02 [JF295260.1] from arid soils | 93 | KF692622 |
| **26** | P2-52 | 588 | Uncultured eubacterium Hb7-K2 [AJ298575.1] from rosy discolouration of masonry and lime wall paintings | 99 | KF692623 |
| **27** | P2-39 | 582 | Uncultured bacterium, clone ncd2161f11c1 [JF185740.1] from skin, popliteal fossa of Homo sapiens | 96 | KF692624 |
| **28** | P2-75 | 584 | *Actinomycetospora chlora*, strain: TT07I-57 [AB514519.1] from a paddy soil on Iriomote Island, Okinawa, Japan | 98 | KF692625 |
| **29** | P2-11 | 583 | *Nocardioides* sp. Gsoil BX5-10 [GQ339904.1] from soil of a ginseng field | 96 | KF692626 |
| **30** | P3-29 | 580 | Uncultured actinobacterium*,* clone FBP483 [AY250887.1] from lichen-dominated Antarctic: Southern Victoria Land, McMurdo Dry Valleys | 99 | KF692702 |
| **31** | P3-27 | 588 | Uncultured actinobacterium*,* clone E6 [KC442786.1] from soils of the Lake Wellman Area, Darwin Mountains, South Victoria Land, Antarctica | 99 | KF692707 |
| **32** | P3-51 | 588 | Uncultured eubacterium MP1-K4 [AJ298571.1] from rosy discolouration of masonry and lime wall paintings | 99 | KF692627 |
| **33** | P3-47 | 588 | Uncultured actinobacterium*,* clone E6 [KC442786.1] from soils of the Lake Wellman Area, Darwin Mountains, South Victoria Land, Antarctica | 99 | KF692709 |
| **34** | P3-38 | 588 | *Rubrobacteraceae* bacterium, isolate VF70612_S4 [FR852391.1] from a green biofilm | 99 | KF692628 |
| **35** | P3-81 | 588 | Uncultured eubacterium MP1-K6 [AJ298572.1] from rosy discolouration of masonry and lime wall paintings | 99 | KF692629 |
| **36** | P3-80 | 588 | Uncultured eubacterium Hb7-K2 [AJ298575.1] from rosy discolouration of masonry and lime wall paintings | 99 | KF692630 |
| **37** | P3-41 | 588 | Uncultured eubacterium Hb7-K1 [AJ298576.1] from rosy discolouration of masonry and lime wall paintings | 99 | KF692631 |
| **38** | P3-43 | 588 | Uncultured actinobacterium*,* clone FBP483 [AY250887.1] from lichen-dominated Antarctic: Southern Victoria Land, McMurdo Dry Valleys | 99 | KF692708 |
| **39** | P3-72 | 588 | *Rubrobacter* sp. CBF L56 [AB166956.1] from deep-sea sediment, Central Basin Fault, West Philippine Basin | 99 | KF692632 |
| **40** | P3-67 | 588 | Uncultured bacterium, clone Hb6-K1 [AJ400551.1] from two different biodeteriorated wall paintings | 99 | KF692633 |
| **41** | P3-84 | 588 | Uncultured bacterium, clone A16 [AM746694.1] from rosy-discoloured mural wall painting of the Crypt of the Original Sin, Matera, Italy | 98 | KF692634 |
| **42** | R1-48 | 547 | Uncultured bacterium, clone TX4CB_142 [FJ153011.1] from alkaline saline soils of the former lake Texcoco, Mexico | 98 | KF692635 |
| **43** | R1-8 | 531 | Uncultured *Rubrobacter* sp., clone K8 [AM161167.1] from mural paintings in the Chapel of St. Virgil, Vienna, Austria | 100 | KF692636 |
| **44** | R1-11 | 586 | *Jiangella* sp. 13658J [EU741189.1] from marine sediment, Cabo Blanco Absolute Natural, Costa Rica | 97 | KF692637 |
| **45** | R1-43 | 588 | *Rubrobacter bracarensis*, strain VFA70612_S5 [HE672088.1] from a green biofilm of a biodeteriorated monument | 99 | KF692638 |
| **46** | R1-24 | 588 | Uncultured bacterium, clone A7 [AM746685.1] from rosy-discoloured mural wall painting of the Crypt of the Original Sin, Matera, Italy | 99 | KF692639 |
| **47** | R1-29 | 588 | Uncultured gamma proteobacterium, clone B2-K12 [KC535205.1] from clothes from mummies in the Capuchin catacombs in Palermo, Italy | 99 | KF692640 |
| **48** | R1-26 | 570 | *Janibacter corallicola*, strain: 02PA-Ca-009 [AB286024.1] from a coral in Palau | 98 | KF692641 |
| **49** | R1-27 | 584 | Uncultured actinobacterium, clone SC6-RK112 [HF584649.1] from white spots on mural paintings from Etruscan tombs | 96 | KF692642 |
| **50** | R1-6 | 569 | Uncultured bacterium, clone TX4CB_142 [FJ153011.1] from alkaline saline soils of the former lake Texcoco, Mexico | 98 | KF692668 |
| **51** | R1-42 | 583 | Uncultured bacterium, clone P-11_B21 [HQ910321.1] from desert soil in the Mars Desert Research Station, Utah, USA. | 97 | KF692669 |
| **52** | R1-28 | 572 | Uncultured *Pseudonocardia* sp., clone 343G [AY571815.1] from hydrocarbon-contaminated soil around Scott Base, Southern Victoria Land, Antarctica | 99 | KF692670 |
| **53** | R1-21 | 566 | Uncultured bacterium, clone CAR-BSb-E9 [FN298047.1] from vertical calcarenite walls of underground tombs from Roman Necropolis of Carmona, Seville, Spain. | 99 | KF692671 |
| **54** | R2-9 | 587 | *Ralstonia insidiosa,* strain BGR27 [KC789786.1] from soil of Shule river, Gansu province, China | 99 | KF692672 |
| **55** | R2-26 | 587 | Uncultured bacterium, clone:13C-M8 [AB205724.1] from denitrifying activated sludge, Ibaraki, Japan | 100 | KF692673 |
| **56** | R2-35 | 588 | *Planococcus* sp. ljh-25 [GU217715.1] from mud volcano 1281 meters above sea, WuSu, Xinjiang, China | 99 | KF692674 |
| **57** | R2-33 | 589 | *Bacillus agaradhaerens*, strain IB-S7 [FN432808.1] from mud from salt lake, Buryatiya, Russia | 99 | KF692675 |
| **58** | R2-14 | 588 | *Rubrobacter bracarensis*, strain VFA70612_S4 [HE672087.1] from a green biofilm of a biodeteriorated monument | 99 | KF692676 |
| **59** | R2-13 | 588 | Uncultured *Rubrobacter* sp., clone B8-K12 [KC535155.1] from wall with purple stains in the Capuchin catacombs in Palermo, Italy | 99 | KF692677 |
| **60** | R2-5 | 587 | Uncultured eubacterium Hb7-K1 [AJ298576.1] from rosy discolouration of masonry and lime wall paintings | 99 | KF692678 |
| **61** | R2-34 | 588 | Uncultured eubacterium Hb7-K2 [AJ298575.1] from rosy discolouration of masonry and lime wall paintings | 99 | KF692679 |
| **62** | R2-43 | 589 | *Natribacillus halophilus* [AB449109.1] from soil in Saitama, Okabe, Japan | 98 | KF692680 |
| **63** | R2-48 | 564 | *Thermocrispum agreste*, strain DSM 44070 [NR_026171.1] from waste and mushroom composts | 94 | KF692681 |
| **64** | R2-16 | 583 | *Saccharopolyspora salina*, strain YIM 91168 [EF687715.1] from a salt lake in Xinjiang Province, North-West China | 99 | KF692682 |
| **65** | R2-29 | 576 | Uncultured actinobacterium, clone B6-K26 [KC535176.1] from rosy discolored wall in the Capuchin catacombs in Palermo, Italy | 99 | KF692683 |
| **66** | R3-14 | 580 | *Rubrobacteraceae* bacterium, isolate VF70612_S1 [FR852390.1] from green biofilm | 99 | KF692684 |
| **67** | R3-35 | 580 | Uncultured eubacterium Hb7-K2 [AJ298575.1] from rosy discolouration of masonry and lime wall paintings | 99 | KF692685 |
| **68** | R3-13 | 588 | *Rubrobacter* sp. C05_TS_X24_S3 [EU512989.1] from biodeteriorated monuments:  Necropolis of Carmona, Seville, Spain | 98 | KF692686 |
| **69** | R3-40 | 588 | Uncultured actinobacterium*,* clone FBP483 [AY250887.1] from lichen-dominated Antarctic: Southern Victoria Land, McMurdo Dry Valleys | 99 | KF692687 |
| **70** | R3-12 | 588 | Uncultured *Rubrobacter* sp., clone B7-K27 [KC535229.1] from salt efflorescence in the Capuchin catacombs in Palermo, Italy | 99 | KF692688 |
| **71** | R3-17 | 588 | Uncultured actinobacterium*,* clone B4-K48 [KC535199.1] from hairs of mummies in the Capuchin catacombs in Palermo, Italy | 99 | KF692689 |
| **72** | R3-19 | 588 | Uncultured bacterium, clone A6 [AM746684.1] from rosy-discoloured mural wall painting of the Crypt of the Original Sin, Matera, Italy | 99 | KF692690 |
| **73** | R3-26 | 588 | Uncultured eubacterium Hb7-K2 [AJ298575.1] from rosy discolouration of masonry and lime wall paintings | 99 | KF692691 |
| **74** | R3-20 | 588 | Uncultured eubacterium Hb7-K1 [AJ298576.1] from rosy discolouration of masonry and lime wall paintings | 99 | KF692692 |
| **75** | R3-16 | 588 | Uncultured bacterium, clone A4 [AM746682.1] from rosy-discoloured mural wall painting of the Crypt of the Original Sin, Matera, Italy | 98 | KF692693 |
| **76** | R3-18 | 571 | Uncultured bacterium, clone RamatNadiv03g07 [JF295210.1] from arid soils | 92 | KF692694 |
| **77** | W1-95 | 390 | Uncultured *Rubrobacter* sp., clone K8 [AM161167.1] from mural paintings in the Chapel of St. Virgil, Vienna, Austria | 100 | KF692643 |
| **78** | W1-36 | 582 | Uncultured eubacterium Hb7-K3 [AJ298577.1] from rosy discolouration of masonry and lime wall paintings | 98 | KF692644 |
| **79** | W1-5 | 588 | Uncultured actinobacterium*,* clone F15cmFL477 [JN002718.1] from serpentinized dunite of ultramafic rocks in the Leka ophiolite complex | 98 | KF692645 |
| **80** | W1-37 | 588 | *Rubrobacter* sp. C05_TS_X24_S3 [EU512989.1] from biodeteriorated monuments: Necropolis of Carmona, Seville, Spain | 95 | KF692646 |
| **81** | W1-4 | 586 | Uncultured bacterium, clone A16 [AM746694.1] from rosy-discoloured mural wall painting of the Crypt of the Original Sin, Matera, Italy | 99 | KF692647 |
| **82** | W1-39 | 588 | Uncultured bacterium, clone A3 [AM746681.1] from rosy-discoloured mural wall painting of the Crypt of the Original Sin, Matera, Italy | 98 | KF692648 |
| **83** | W1-92 | 586 | Uncultured bacterium, clone A16 [AM746694.1] from rosy-discoloured mural wall painting of the Crypt of the Original Sin, Matera, Italy | 99 | KF692649 |
| **84** | W1-55 | 588 | *Rubrobacter xylanophilus*, strain DSM 9941 [NR_074552.1] | 94 | KF692650 |
| **85** | W1-81 | 560 | Uncultured bacterium, clone Hb6-K1 [AJ400551.1] from two different biodeteriorated wall paintings | 100 | KF692651 |
| **86** | W2-53 | 588 | Uncultured actinobacterium*,* clone F15cmL164 [JN002746.1] from serpentinized dunite of ultramafic rocks in the Leka ophiolite complex | 99 | KF692652 |
| **87** | W2-14 | 587 | Uncultured actinobacterium*,* clone E6 [KC442786.1] from soils of the Lake Wellman Area, Darwin Mountains, South Victoria Land, Antarctica | 99 | KF692653 |
| **88** | W2-23 | 587 | Uncultured actinobacterium*,* clone F15cmL169 [JN002750.1] from serpentinized dunite of ultramafic rocks in the Leka ophiolite complex | 99 | KF692654 |
| **89** | W2-22 | 587 | Uncultured eubacterium Hb7-K1 [AJ298576.1] from rosy discolouration of masonry and lime wall paintings | 99 | KF692655 |
| **90** | W2-80 | 588 | *Rubrobacter bracarensis*, strain VFA70612_S4 [HE672087.1] from a green biofilm of a biodeteriorated monument | 99 | KF692656 |
| **91** | W2-44 | 419 | Uncultured *Rubrobacter* sp., clone K7 [AM161166.1] from mural paintings in the Chapel of St. Virgil, Vienna, Austria | 100 | KF692657 |
| **92** | W2-6 | 588 | Uncultured actinobacterium*,* clone F15cmFL453 [JN002699.1] from serpentinized dunite of ultramafic rocks in the Leka ophiolite complex | 99 | KF692658 |
| **93** | W2-62 | 579 | Uncultured bacterium, clone A6 [AM746684.1] from rosy-discoloured mural wall painting of the Crypt of the Original Sin, Matera, Italy | 99 | KF692603 |
| **94** | W2-33 | 588 | Uncultured bacterium, clone A7 [AM746685.1] from rosy-discoloured mural wall painting of the Crypt of the Original Sin, Matera, Italy | 99 | KF692604 |
| **95** | W2-10 | 588 | *Rubrobacter* sp. CBF L56 [AB166956.1] from deep-sea sediment, Central Basin Fault, West Philippine Basin | 99 | KF692605 |
| **96** | W2-96 | 588 | Uncultured *Rubrobacter* sp., clone K8 [AM161167.1] from mural paintings in the Chapel of St. Virgil, Vienna, Austria | 100 | KF692606 |
| **97** | W2-64 | 588 | Uncultured bacterium, clone Hb6-K1 [AJ400551.1] from two different biodeteriorated wall paintings | 100 | KF692607 |
| **98** | W3-95 | 580 | Uncultured bacterium, clone Hb6-K1 [AJ400551.1] from two different biodeteriorated wall paintings | 100 | KF692608 |
| **99** | W3-76 | 587 | Uncultured bacterium, clone WT42H53 [HE966153.1] from composting soil | 100 | KF692609 |
| **100** | W3-58 | 586 | Uncultured bacterium, clone A3 [AM746681.1] from rosy-discoloured mural wall painting of the Crypt of the Original Sin, Matera, Italy | 99 | KF692610 |
| **101** | W3-86 | 588 | *Rubrobacter bracarensis*, strain VFA70612_S5 [HE672088.1] from a green biofilm of a biodeteriorated monument | 99 | KF692695 |
| **102** | W3-62 | 588 | Uncultured eubacterium Hb7-K1 [AJ298576.1] from rosy discolouration of masonry and lime wall paintings | 99 | KF692696 |
| **103** | W3-89 | 588 | Uncultured bacterium, clone Hb6-K1 [AJ400551.1] from two different biodeteriorated wall paintings | 100 | KF692697 |
| **104** | W3-15 | 588 | Uncultured bacterium, clone A16 [AM746694.1] from rosy-discoloured mural wall painting of the Crypt of the Original Sin, Matera, Italy | 98 | KF692698 |
| **105** | W3-30 | 583 | *Saccharopolyspora salina,* strain YIM 91168 [EF687715.1] from a salt lake in Xinjiang Province, North-West China | 99 | KF692699 |
| **106** | W3-6 | 576 | Uncultured actinobacterium*,* clone B6-K26 [KC535176.1] from rosy discolored wall in the Capuchin catacombs in Palermo, Italy | 99 | KF692700 |
| **107** | W3-46 | 583 | Uncultured bacterium, clone P11-P81 [GU574055.1] from mould-colonized water damaged building material | 99 | KF692701 |
