# Supplementary material for: Halophilic Microorganisms Are Responsible for the Rosy Discolouration of Saline Environments in Three Historical Buildings with Mural Paintings
Source: PLoS One. 2014 Aug 1;9(8):e103844. doi: 10.1371/journal.pone.0103844 (PMC4118916; doi:10.1371/journal.pone.0103844)
Supplement: Table S4 — Phylogenetic affiliations of the archaeal sequences. Phylogenetic affiliations of the partial 16S rRNA gene sequences obtained from all archaeal clones in the samples from the three buildings. Accession codes: Sequences were deposited at the NCBI GenBank under the accession numbers KF692550–KF692709 for the cloned sequences. (DOCX) [file pone.0103844.s005.docx]

**Supporting Table S4.** **Phylogenetic affiliations of the archaeal sequences.** Phylogenetic affiliations of the partial 16S rRNA gene sequences obtained from all archaeal clones in the samples from the three buildings. Accession codes: Sequences were deposited at the NCBI GenBank under the accession numbers KF692550-KF692709 for the cloned sequences.

| **DGGE band** | **Clone number** | **Sequence length [bp]** | **Nearest published relative and isolation source from NCBI database** | **Similarity (%)** | **Accession number** |
| --- | --- | --- | --- | --- | --- |
| **1** | P2-16 | 552 | Uncultured archaeon*,* clone TP-AM-A17 [HQ645127.1] from soil samples from high altitude meadow, China | 99 | KF692550 |
| **2** | P2-9 | 552 | Uncultured archaeon, clone PM37 [AJ608183.1] from soil, Netherlands | 99 | KF692551 |
| **3** | P2-10 | 447 | Uncultured *Halococcus* sp., clone 16 [FN435871.1] from salt efflorescence on medieval stonework in Chapel of St. Virgil, Vienna, Austria | 99 | KF692552 |
| **4** | P2-36 | 552 | Uncultured archaeon, clone sscpArc_V5A5 [FM179191.1] from groundwater, Tyrol, Austria | 98 | KF692553 |
| **5** | P2-5 | 552 | Uncultured archaeon*,* clone DOL-A146 [JF737821.1] from dolomite rock from the Nanjiang Canyon, Guizhou Karst Region, China | 98 | KF692554 |
| **6** | P2-15 | 555 | Uncultured *Halobacterium* sp., clone A9-K51 [KC535277.1] from wall samples in the Capuchin catacombs in Palermo, Italy | 99 | KF692555 |
| **7** | P2-17 | 555 | Unidentified archaeon H6-K6 [AJ291422.1] from two disparate deteriorated ancient wall paintings | 99 | KF692556 |
| **8** | P2-18 | 555 | *Halococcus hamelinensis* 100A6 [NR_028168.1] from stromatolites of Shark Bay, Hamelin Pool, Western Australia | 98 | KF692557 |
| **9** | P2-45 | 555 | Uncultured *Halobacterium* sp., clone library K2, clone 79 [FN433757.1] from salt efflorescence on medieval stonework in Chapel of St. Virgil, Vienna, Austria | 99 | KF692558 |
| **10** | P2-19 | 555 | Uncultured *Halobacterium* sp., clone K14 [AM159641.1] from salt efflorescence on medieval stonework in Chapel of St. Virgil, Vienna, Austria | 99 | KF692559 |
| **11** | P2-14 | 555 | Unidentified archaeon*,* H6-K5 [AJ291421.1] from two disparate deteriorated ancient wall paintings | 99 | KF692560 |
| **12** | P2-13 | 555 | Uncultured *Halococcus* sp., clone A9-K64 [KC535280.1] from wall samples in the Capuchin catacombs in Palermo, Italy | 98 | KF692561 |
| **13** | P2-48 | 555 | Uncultured *Halococcus* sp., clone library K9, clone 2 [FN435859.1] from salt efflorescence on medieval stonework in Chapel of St. Virgil, Vienna, Austria | 99 | KF692562 |
| **14** | P3-21 | 555 | *Halococcus* sp. FC211 [EU308208.1] from a solar saltern in Western Greece | 98 | KF692563 |
| **15** | P3-48 | 555 | Uncultured *Halobacterium* sp., clone A9-K41 [KC535276.1] from wall samples in the Capuchin catacombs in Palermo, Italy | 99 | KF692564 |
| **16** | P3-37 | 555 | Uncultured *Halococcus* sp., clone 3 [FN435860.1] from salt efflorescence on medieval stonework in Chapel of St. Virgil, Vienna, Austria | 98 | KF692565 |
| **17** | P3-14 | 555 | Uncultured *Halobacterium* sp., clone K14 [AM159641.1] from salt efflorescence on medieval stonework in Chapel of St. Virgil, Vienna, Austria | 99 | KF692566 |
| **18** | P3-36 | 554 | *Halococcus* sp. KeC-02 [AB534723.1] from a seawater aquarium, Nakano-ku, Minamidai, Tokyo, Japan | 98 | KF692567 |
| **19** | P3-26 | 555 | *Halococcus* sp. IARI-ABCL-7 [JX428954.1] from saline sediments and water, Chilka Lake, Orrisa, India | 98 | KF692568 |
| **20** | P3-38 | 555 | *Halococcus* sp. KeC-16 [AB534732.1] from a seawater aquarium, Nakano-ku, Minamidai, Tokyo, Japan | 98 | KF692569 |
| **21** | P3-9 | 555 | Haloarchaeon Nie 13 [AB291223.1] from salt field soil in Nie, Ishikawa, Japan | 98 | KF692570 |
| **22** | R1-102 | 555 | *Halalkalicoccus jeotgali B3* [NR_102920.1] from salt-fermented seafood, South Korea | 99 | KF692585 |
| **23** | R1-99 | 555 | Uncultured *Halobacterium* sp., clone A9-K41 [KC535276.1] from wall samples in the Capuchin catacombs in Palermo, Italy | 99 | KF692586 |
| **24** | R1-98 | 555 | Uncultured *Halococcus* sp., clone 3 [FN435860.1] from salt efflorescence on medieval stonework in Chapel of St. Virgil, Vienna, Austria | 98 | KF692587 |
| **25** | R1-104 | 554 | *Halococcus* sp. IARI-ABCL-7 [JX428954.1] from saline sediments and water, Chilka Lake, Orrisa, India | 98 | KF692588 |
| **26** | R1-43 | 555 | Halococcus sp. KeC-08 [AB534726.1] from a seawater aquarium, Nakano-ku, Minamidai, Tokyo, Japan | 98 | KF692589 |
| **27** | R2-6 | 555 | *Halalkalicoccus* sp. C15 [DQ373058.1] from Salda Lake, Turkey | 99 | KF692590 |
| **28** | R2-19 | 555 | Uncultured archaeon*,* clone FR2_c49a [GU126495.1] from spacecraft assembly clean room, Germany | 97 | KF692591 |
| **29** | R2-42 | 555 | *Halalkalicoccus* sp. YIM 93701 [JF449426.1] from salt soil of Lup Nur region, Xinjiang Province, North-West China | 99 | KF692592 |
| **30** | R2-1 | 589 | Uncultured archaeon*,* clone FlD_6_EA [JX865727.1] from intensive care unit floor, Regensburg, Germany | 99 | KF692593 |
| **31** | R2-17 | 554 | Uncultured *Halococcus* sp., clone 35 [FN435865.1] from salt efflorescence on medieval stonework in Chapel of St. Virgil, Vienna, Austria | 95 | KF692594 |
| **32** | R2-20 | 555 | *Natronorubrum* sp., strain CG-4 [FN376860.1] from sediment of saline Lake Chagannor in Inner Mongolia, China | 99 | KF692595 |
| **33** | R2-22 | 555 | *Halococcus* sp. CBA1101 [JX989265.1] from marine environment in South Korea | 99 | KF692596 |
| **34** | R3-46 | 574 | *Halalkalicoccus* sp. C15 [DQ373058.1] from Salda Lake, Turkey | 98 | KF692597 |
| **35** | R3-28 | 555 | *Halalkalicoccus* sp. YIM 93701 [JF449426.1] from salt soil of Lup Nur region, Xinjiang Province, North-West China | 99 | KF692598 |
| **36** | R3-36 | 555 | *Halococcus hamelinensis* 100A6 [NR_028168.1] from stromatolites of Shark Bay, Hamelin Pool, Western Australia | 98 | KF692599 |
| **37** | R3-34 | 555 | Unidentified archaeon H6-K5 [AJ291421.1] from two disparate deteriorated ancient wall paintings | 98 | KF692600 |
| **38** | R3-45 | 555 | Uncultured archaeon, clone 24 [FN435863.1] from salt efflorescence on medieval stonework in Chapel of St. Virgil, Vienna, Austria | 98 | KF692601 |
| **39** | R3-15 | 555 | Uncultured *Halobacterium* sp., clone A9-K63 [KC535279.1] from wall samples in the Capuchin catacombs in Palermo, Italy | 97 | KF692602 |
| **40** | W1-22 | 555 | *Halococcus morrhuae*, clone K16 [AM159638.1] ] from salt efflorescence on medieval stonework in Chapel of St. Virgil, Vienna, Austria | 98 | KF692571 |
| **41** | W1-5 | 555 | Unidentified archaeon H6-K5 [AJ291421.1] from two disparate deteriorated ancient wall paintings | 99 | KF692572 |
| **42** | W1-6 | 555 | *Halococcus* sp. IARI-ABCL-7 [JX428954.1] from saline sediments and water, Chilka Lake, Orrisa, India | 98 | KF692573 |
| **43** | W1-16 | 555 | Uncultured *Halococcus* sp., clone A9-K64 [KC535280.1] from wall samples in the Capuchin catacombs in Palermo, Italy | 97 | KF692574 |
| **44** | W2-71 | 555 | Uncultured archaeon*,* clone FR2_c49a [GU126495.1] from spacecraft assembly clean room, Germany | 99 | KF692575 |
| **45** | W2-30 | 555 | *Halalkalicoccus* sp. C15 [DQ373058.1] from Salda Lake, Turkey | 99 | KF692576 |
| **46** | W2-39 | 555 | Uncultured archaeon*,* clone FlD_6_EA [JX865727.1] from intensive care unit floor, Regensburg, Germany | 99 | KF692577 |
| **47** | W2-20 | 555 | *Halococcus* sp. IARI-ABCL-7 [JX428954.1] from saline sediments and water, Chilka Lake, Orrisa, India | 98 | KF692578 |
| **48** | W2-33 | 555 | Uncultured *Halalkalicoccus* sp., clone 97 [FN433769.1] from salt efflorescence on medieval stonework in Chapel of St. Virgil, Vienna, Austria | 98 | KF692579 |
| **49** | W3-2 | 555 | Uncultured *Halococcus* sp., clone library K9, clone 2 [FN435859.1] from salt efflorescence on medieval stonework in Chapel of St. Virgil, Vienna, Austria | 99 | KF692580 |
| **50** | W3-1 | 554 | *Halococcus* sp. KeC-16 [AB534732.1] from a seawater aquarium, Nakano-ku, Minamidai, Tokyo, Japan | 98 | KF692581 |
| **51** | W3-97 | 555 | Uncultured *Halobacterium* sp., clone K14 [AM159641.1] from salt efflorescence on medieval stonework in Chapel of St. Virgil, Vienna, Austria | 99 | KF692582 |
| **52** | W3-100 | 555 | Uncultured *Halobacterium* sp., clone A9-K51 [KC535277.1] from wall samples in the Capuchin catacombs in Palermo, Italy | 99 | KF692583 |
| **53** | W3-98 | 553 | Unidentified archaeon H6-K5 [AJ291421.1] from two disparate deteriorated ancient wall paintings | 99 | KF692584 |
